# Missense mutations of the ephrin receptor EPHA1 associated with Alzheimer’s disease disrupt receptor signaling functions

**DOI:** 10.1016/j.jbc.2024.108099

**Published:** 2024-12-18

**Authors:** Mike Matsumoto, Maricel Gomez-Soler, Sara Lombardi, Bernhard C. Lechtenberg, Elena B. Pasquale

**Affiliations:** 1 Cancer Center, Sanford Burnham Prebys Medical Discovery Institute, La Jolla, California, USA

**Keywords:** Alzheimer’s disease, receptor tyrosine kinase, Eph receptor, receptor modification, phosphotyrosine, phosphoserine, N-linked glycosylation, proteolytic cleavage

## Abstract

Missense mutations in the EPHA1 receptor tyrosine kinase have been identified in Alzheimer’s patients. To gain insight into their potential role in disease pathogenesis, we investigated the effects of four of these mutations. We show that the P460L mutation in the second fibronectin type III (FN2) domain drastically reduces EPHA1 cell surface localization while increasing tyrosine phosphorylation of the cell surface–localized receptor. The R791H mutation in the kinase domain abolishes EPHA1 tyrosine phosphorylation, indicating abrogation of kinase-dependent signaling. Furthermore, both mutations decrease EPHA1 phosphorylation on S906 in the kinase-SAM linker region, suggesting impairment of a noncanonical form of signaling regulated by serine/threonine kinases. The R492Q mutation, also in the FN2 domain, has milder effects than the P460L mutation while the R926C mutation in the SAM domain increases S906 phosphorylation. We also found that EPHA1 undergoes constitutive proteolytic cleavage in the FN2 domain, generating a soluble 55 kDa N-terminal fragment containing the ligand-binding domain and a transmembrane 60 kDa C-terminal fragment. The 60 kDa WT fragment is phosphorylated on both tyrosine residues and S906, suggesting signaling functions. The P460L mutant 60 kDa fragment undergoes proteasomal degradation and the R791H mutant fragment lacks tyrosine phosphorylation and has decreased S906 phosphorylation. These findings advance our understanding of EPHA1 signaling mechanisms and support the notion that alterations in EPHA1 signaling due to missense mutations contribute to Alzheimer’s disease pathogenesis.

Numerous studies have linked common single nucleotide polymorphisms (SNPs) near the *EPHA1* coding region with the risk of late-onset Alzheimer’s disease, but the effects of these SNPs remain poorly understood (1, 2, 3, 4, 5, 6). Although association of these SNPs with *EPHA1* expression in immune cells has been reported (7, 8), suggesting a role in neuroinflammation, more recent studies show effects on the expression of neighboring genes such as *EPHA1-AS1* and *ZYX* (Zyxin) in blood myeloid cells (7, 9, 10, 11, 12). Thus, the SNPs may play a role in Alzheimer’s disease by affecting other genes besides *EPHA1*. Nevertheless, the attention on *EPHA1* triggered by the SNPs led to recent searches for missense mutations in the *EPHA1* coding sequence in different cohorts of late-onset Alzheimer’s patients. These efforts identified eight rare variants encoding missense mutations potentially associated with the disease (13, 14, 15, 16) (Table S1 and Fig. S1). Moreover, a recent study suggests that the EPHA1 V160A and M900V common variants (uniprot.org) may have protective effect in small vessel ischemic disease, a pathology frequently associated with late-onset Alzheimer’s disease (17). These missense mutations suggest a potential role of EPHA1 in Alzheimer’s disease, highlighting the need to characterize their functional effects.

EPHA1 is the founding member of the large Eph family of receptor tyrosine kinases, which comprises 14 members (18, 19). However, it is one of the least characterized Eph receptors and there is limited information about its physiological and pathological activities. EPHA1 signaling, involving tyrosine phosphorylation and kinase activity, can be induced by glycosylphosphatidylinositol-linked ephrinA ligands anchored on the surface of neighboring cells or released in soluble form (19, 20, 21, 22, 23). In addition, EPHA1 contains three phosphorylation sites (S906, S908, and S910; phosphosite.org) in the linker region that connects the kinase domain with the C-terminal sterile-alpha motif (SAM) domain. These three phosphorylation sites are conserved in EPHA1 and EPHA2 among the Eph receptors (phosphosite.org). Their signaling function in EPHA1 has not been characterized, but in the case of EPHA2, it is well established that phosphorylation of the kinase-SAM linker region by serine/threonine kinases mediates a noncanonical form of signaling that is independent of ligand binding and kinase activity (24, 25, 26). Best characterized among these EPHA2 phosphosites is S897, corresponding to EPHA1 S906, which can be phosphorylated by RSK, AKT, and PKA (24, 27, 28) leading to cell resistance to stress, cell migration, and cancer progression (29, 30). Here we show that missense mutations identified in Alzheimer’s patients disrupt EPHA1 signaling ability and thus may play a role in disease pathology.

## Results

### EPHA1 missense mutations identified in Alzheimer’s patients

A search for mutations in the *EPHA1* coding sequence in three independent cohorts of late-onset Alzheimer’s patients of Hispanic and European ancestry identified seven rare variants encoding missense mutations potentially associated with the disease, including R337Q, P460L, R471Q, V514I, R791H, H888Y, and R926C (13) (Table S1 and Fig. S1). Of these, P460L was the most significantly associated with the disease, since it was identified in all three cohorts and showed complete segregation with the disease in four individuals from an extended family. The six other EPHA1 nonsynonymous coding variants were nominally associated with late-onset Alzheimer’s disease at *p* > 0.05, with the R791H mutation identified in three families and the others in one or two patients. Three other independent studies identified an additional *EPHA1* rare variant encoding the R492Q mutant as potentially linked to Alzheimer’s disease (14, 15, 16).

As a member of the Eph receptor family, the extracellular portion of EPHA1 includes an N-terminal ligand-binding domain, a cysteine-rich region comprising a Sushi domain and an EGF-like domain, and two fibronectin type III domains (Fig. S1, left). The cytoplasmic portion of EPHA1 includes a juxtamembrane segment, the tyrosine kinase domain, and a SAM domain connected to the kinase domain through a short linker of ∼ 20 amino acids (31). EPHA1 lacks the C-terminal tail that is present in other Eph receptors, including the PDZ domain-binding motif.

The eight EPHA1 missense mutations identified in Alzheimer's patients are located in different EPHA1 domains, including one in the first fibronectin type III (FN1) domain, four in the second fibronectin type III (FN2) domain, two in the kinase domain, and one in the SAM domain (Table S1; Figs. S1 and S2*A*). Analysis using the Poly-Phen2 program (32) (genetics.bwh.harvard.edu/pph2/) predicts that the P460L mutation in the FN2 domain, the R791H mutation in the kinase domain, and the R926C mutation in the SAM domain are likely to drastically alter EPHA1 function (Fig. S1, right), and that the R492Q mutation in the FN2 domain may also have functional significance. In contrast, the R337Q, R471Q, V514I, and H888Y mutations are not predicted to substantially affect EPHA1 functional properties. Therefore, we focused on the P460L, R492Q, R791H, and R926C mutations.

### The EPHA1 P460L mutant exhibits impaired N-glycosylation

**Figure 1:**
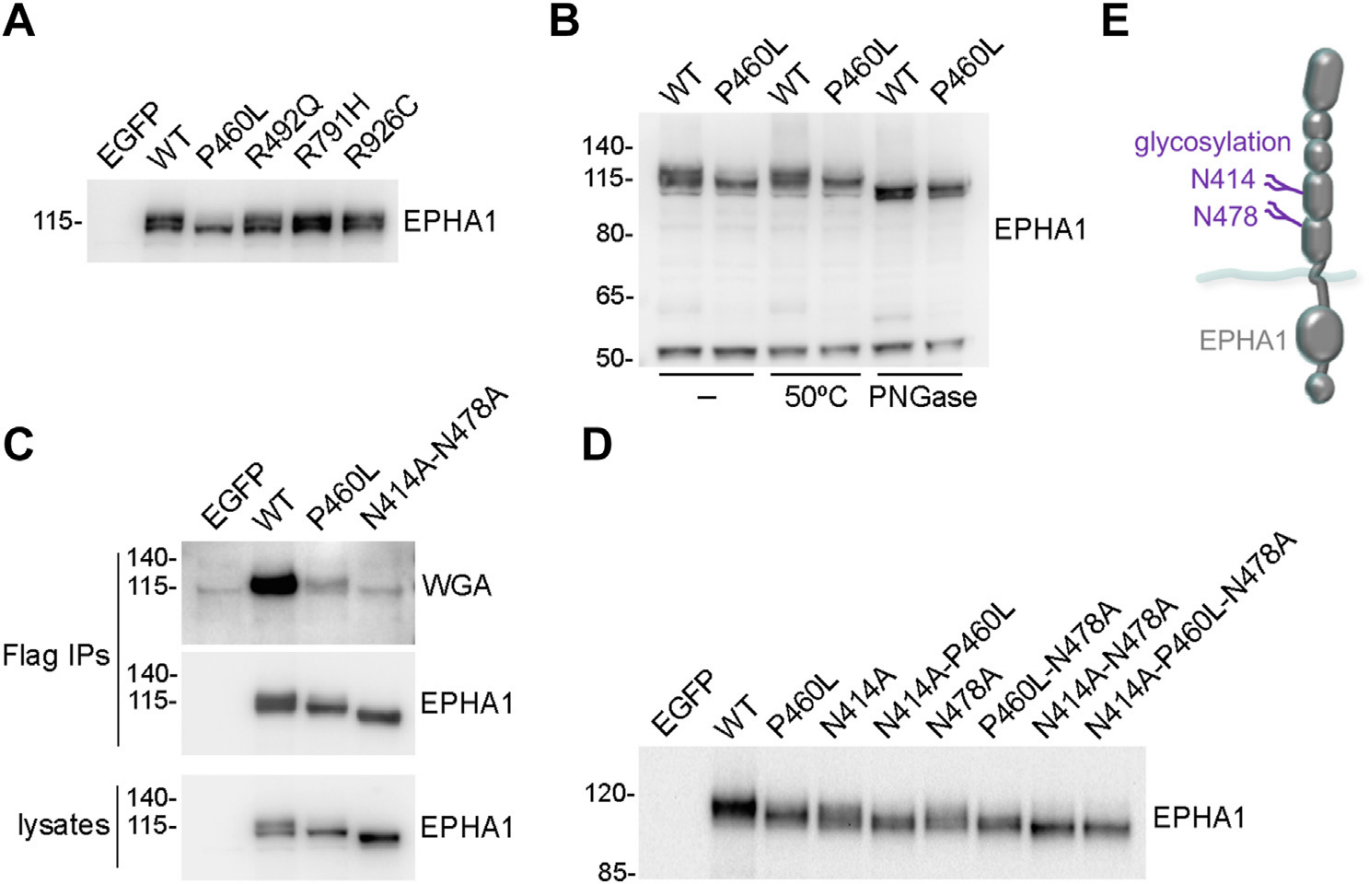
**The fully N-glycosylated form of EPHA1 is greatly reduced by the P460L mutation.** HEK293 cells were transiently transfected with constructs encoding EGFP, EPHA1 WT, or the indicated EPHA1 mutants. *A*, cell lysates were probed by immunoblotting with the EPHA1 SAM antibody. *B*, cell lysates were immediately frozen (−) or incubated at 50 °C without or with Peptide:N-glycosidase F (PNGase F). The immunoblots were probed for EPHA1. *C*, Flag immunoprecipitates (IPs) and cell lysates were probed with WGA conjugated to HRP or by immunoblotting for EPHA1. *D*, cell lysates were probed by immunoblotting for EPHA1. *E*, schematic showing the positions of the two identified EPHA1 N-glycosylation sites.

To study the possible functional effects of the four selected mutations identified in Alzheimer’s patients, we transiently expressed in HEK293 cells WT and mutant human EPHA1 with an N-terminal Flag tag. By immunoblotting with a new polyclonal antibody that we generated, which specifically recognizes the EPHA1 C-terminal region including the SAM domain (Fig. S3*A*), we detected two EPHA1 bands with slightly different apparent molecular weights (Fig. 1*A*). The two bands have similar intensity in immunoblots of EPHA1 WT and three of the mutants examined, whereas the lower band is predominant in the case of the P460L mutant.

Since N-linked glycosylation typically accounts for 10 to 20 kDa of the apparent size of an Eph receptor (33, 34), we hypothesized that the EPHA1 upper band may represent the fully glycosylated form while the lower band may be deficient in N-glycosylation (35). Treatment with Peptide:N-glycosidase F (PNGase F) to remove N-linked oligosaccharides indeed decreased the apparent molecular weight of the EPHA1 upper band by 10 to 20 kDa (Fig. 1*B*). It also decreased the size of the lower band, but to a lesser extent, indicating that the lower band contains fewer or shorter oligosaccharide chains. The deglycosylated EPHA1 WT and P460L mutant have similar size, confirming that the difference between WT and mutant bands is due to N-glycosylation.

We next used wheat germ agglutinin (WGA), a lectin that we previously found to bind glycosylated Eph receptors (33), to detect glycosylation of immunoprecipitated EPHA1 (Fig. 1*C*). WGA preferentially labeled the EPHA1 upper band (Fig. 1*C*), suggesting that this form of EPHA1 contains complex oligosaccharide chains with terminal sialic acid, a sugar that binds to WGA. In contrast, the EPHA1 lower band does not bind WGA, as expected for an immature not fully glycosylated form. WGA yielded only a faint signal for the P460L mutant (Fig. 1*C*), consistent with the small amount of the upper band in this mutant.

EPHA1 extracellular asparagines that are part of the N-glycosylation motif NX(S/T) include N59, N338, N414, and N478. Of these, N414 in the FN1 domain and N478 in the FN2 domain are predicted as the most likely N-glycosylation sites (https://services.healthtech.dtu.dk/services/NetNGlyc-1.0/) (36). Analysis of EPHA1 N-glycosylation site mutants revealed that the N414A and N478A single mutations decrease the size of both EPHA1 bands, but less than the two mutations combined (Fig. 1*D*). This implies that both N414 and N478 are glycosylated and that the lower band contains shorter oligosaccharide chains at both sites. The P460L mutation markedly reduced the upper band in both EPHA1 N414A and EPHA1 N478A, suggesting effects of the mutation on glycosylation of both sites. Furthermore, an upper band was not detectable in the EPHA1 N414A-N478A mutant lacking both glycosylation sites, as expected if the difference in the size of upper and lower bands is due to N-glycosylation. The EPHA1 N414A-N478A double mutant appears to have a reduced size similar to that of deglycosylated EPHA1 and is not labeled above background by WGA (Fig. 1, *B*–*D*), indicating that EPHA1 N-glycosylation is confined to N414 and N478 (Fig. 1*E*). Furthermore, as expected, the N414A-P460L-N478A triple mutant has a size similar to the N414A-N478A double mutant. These results show that the fully N-glycosylated form of EPHA1, corresponding to the upper band, is greatly reduced as a consequence of the P460L mutation.

### The P460L mutation decreases EPHA1 cell surface localization

**Figure 2:**
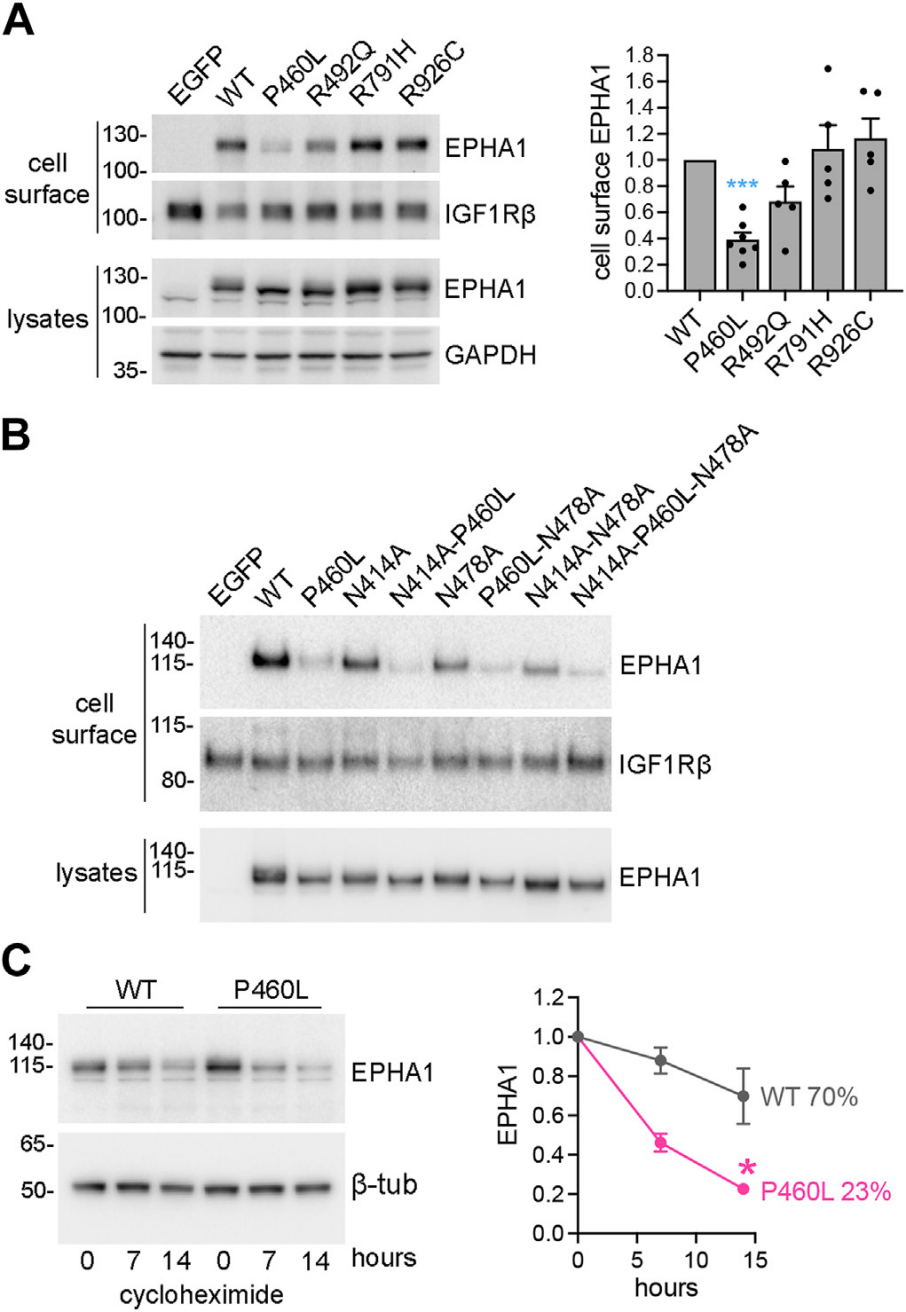
**The P460L mutation and impaired N-glycosylation decrease EPHA1 cell surface localization.***A*, proteins on the surface of HEK293 cells transiently expressing EPHA1 WT or the indicated mutants were biotinylated and pulled down with streptavidin beads. Pulled down proteins (cell surface) and cell lysates were probed as indicated. IGF1 receptor β (IGF1Rβ) was detected as a control that should have similar cell surface localization in all samples. The graph shows cell surface expression of the indicated mutants normalized to EPHA1 WT based on quantification of immunoblots. The bars show means and SE from quantification of five to seven experiments, with the individual data points shown as *black* dots. ∗∗∗, *p* < 0.001 for the comparison with WT using one-way ANOVA and Dunnett’s multiple comparisons test. *B*, cell surface biotinylation experiment similar to (A) comparing the indicated mutants. *C*, HEK293 cells stably expressing EPHA1 WT or the P460L mutant were treated with cycloheximide for the indicated times and probed by immunoblotting with the EPHA1 SAM antibody and a β-tubulin antibody as a loading control. The graph shows EPHA1 levels normalized to EPHA1 level at time 0 of cycloheximide treatment, quantified from the immunoblots from four experiments. The error bars represent SEs. ∗, *p* < 0.05 by unpaired Student’s *t* test for the comparison between EPHA1 WT and L460L at the 14 h time point.

Secreted and transmembrane proteins undergo N-glycosylation in the endoplasmic reticulum and Golgi apparatus, during their transport to the cell surface (37). Thus, fully glycosylated EPHA1 (corresponding to the upper band) may be on the cell surface, whereas partially glycosylated EPHA1 (corresponding to the lower band) may represent an immature form that has not yet reached the cell surface. To assess the extent of EPHA1 cell surface localization, we biotinylated cell surface proteins in HEK293 cells transiently or stably transfected with WT or mutant EPHA1 using a membrane-impermeable biotinylation reagent. We then probed the biotinylated proteins, pulled down with streptavidin beads, by immunoblotting for EPHA1. We detected a band of similar intensity for EPHA1 WT and the R791H and R926C mutants, whereas the P460L mutant band was much weaker and the R492Q mutant appeared weaker in some experiments but not others (Fig. 2*A*). This result is consistent with the notion that the upper band, which is much less abundant in the P460L mutant, represents the cell surface–localized fully N-glycosylated form of EPHA1.

Cell surface biotinylation experiments with the EPHA1 N-glycosylation–deficient mutants revealed that the N414A and N478A single mutations also decrease EPHA1 cell surface localization and that two mutations combined have an even greater effect (Fig. 2*B*). This implies that lack of glycosylation on both N414 and N478 impairs EPHA1 trafficking to the cell surface, consistent with prior reports on other transmembrane proteins (38, 39). However, the P460L mutation decreased the cell surface localization of not only the N414A and N478A single mutants, but also the N414A-N478A double mutant. This suggests that the impairment of EPHA1 P460L cell surface localization depends at least in part on factors other than its deficient N-glycosylation. In fact, the P460L mutation seems unlikely to directly affect EPHA1 N-glycosylation, since structural modeling shows that the P460 residue is located at one end of the FN2 domain, near the linker connecting FN2 with the transmembrane helix, whereas N478 is at the opposite end of the FN2 domain and N414 is in a different (FN1) domain (Fig. S2, A and B). We speculate that the deficient N-glycosylation of EPHA1 P460L might be a consequence of impaired trafficking through the endoplasmic reticulum and Golgi apparatus, perhaps due to FN2 domain misfolding caused by the mutation.

Since misfolding would be expected to destabilize the EPHA1 protein decreasing its half-life, we treated HEK293 cells stably expressing EPHA1 WT or P460L mutant with cycloheximide to stop protein synthesis and measured the decrease in EPHA1 expression over time by immunoblotting (Fig. 2*C*). Consistent with a destabilizing effect of the mutation due to misfolding, the cycloheximide treatment shows that EPHA1 P460L is a less stable protein with a shorter half-life than EPHA1 WT. In fact, lower protein expression of EPHA1 P460L than WT have been observed in cells with similar WT and mutant mRNA levels (40).

### EPHA1 can be cleaved into cell-associated and soluble fragments

**Figure 3:**
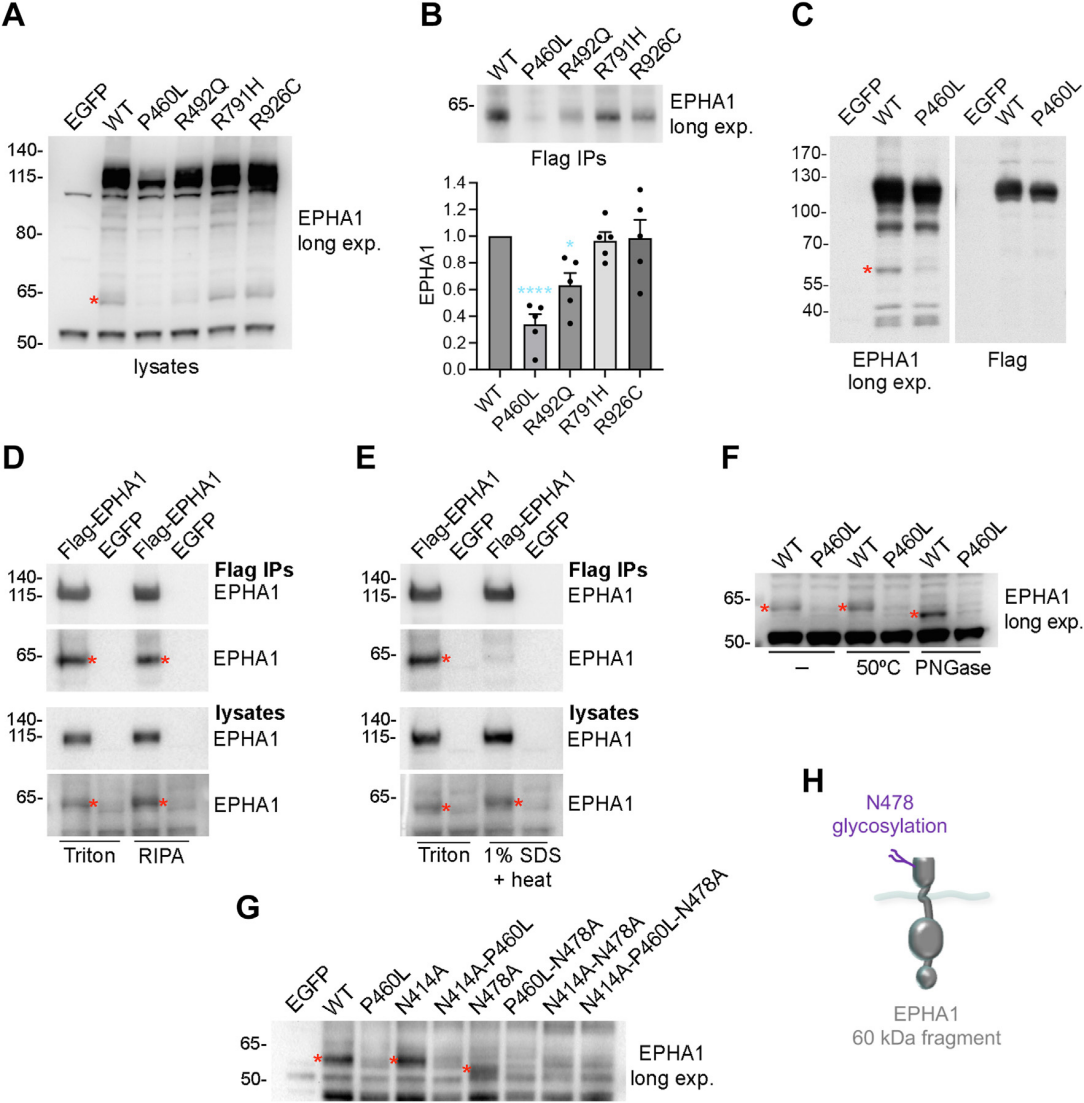
**The P460L mutation reduces the abundance of an EPHA1 60 kDa C-terminal fragment.** HEK293 cells were transiently transfected with constructs encoding EGFP, EPHA1WT, or the indicated EPHA1 mutants. *A*, cell lysates were probed with the EPHA1 SAM domain antibody; the long exposure (long exp.) reveals a fragment of ∼60 kDa in cells expressing EPHA1 WT and some of the mutants. *B*, the 60 kDa band is also detected in Flag immunoprecipitates. The graphs show means and SEs from quantification of five experiments, with the individual data points shown as *black* dots. ∗, *p* < 0.05 and ∗∗∗∗, *p* < 0.0001 for the comparison with EPHA1 WT by one-way ANOVA and Dunnett’s multiple comparison test. *C*, cell lysates were probed with antibodies recognizing the EPHA1 SAM domain or the Flag epitope at the N terminus of EPHA1. *D*, the EPHA1 60 kDa fragment is detected in Flag immunoprecipitates carried out in both 0.5% TX-100 buffer and modified RIPA buffer that contains 0.1% SDS. *E*, the EPHA1 60 kDa fragment is not detected in Flag immunoprecipitates carried out in modified RIPA buffer containing 1% SDS and heated to disrupt protein interactions. EPHA1 immunoblots of Flag immunoprecipitates (Flag IPs) and lysates are shown. *F*, longer exposure of the lower part of the EPHA1 immunoblot shown in Fig. 1B highlights the EPHA1 C-terminal 60 kDa fragment, which exhibits a lower size following deglycosylation. *G*, the 60 kDa fragment has reduced size as a consequence of the N478 mutation that prevents glycosylation at this site. *H*, schematic illustrating the EPHA1 60 kDa C-terminal fragment. A *red* asterisk in (A,C,D,E,F,G) marks the EPHA1 60 kDa fragment.

In addition to full-length EPHA1, the EPHA1 SAM domain antibody also detects a fainter ∼60 kDa band in HEK293 cell lysates (Fig. 3*A*, red asterisk) and Flag immunoprecipitates (Fig. 3*B*). This 60 kDa fragment is drastically reduced in cells expressing the P460L mutant (Fig. 3, *A* and *B*). It also appears to be slightly reduced in cells expressing the R492Q mutant, while its abundance is similar to WT in the case of the R791H and R926C mutants. Although the 60 kDa C-terminal fragment does not contain the Flag tag (Fig. 3*C*), the fragment was nevertheless detected in Flag immunoprecipitates of full-length EPHA1 (Fig. 3, *D* and *E*). Co-immunoprecipitation of the fragment with full-length EPHA1 was observed not only in lysis buffer containing the mild detergent 0.5% TX-100, but also in modified RIPA buffer, which contains several detergents including 0.1% SDS (Fig. 3*D*). Interaction of the 60 kDa fragment with full-length EPHA1 could be disrupted by heating the lysates in modified RIPA buffer containing 1% SDS before immunoprecipitation (Fig. 3*E*), indicating a strong and stable association that is not mediated by a disulfide bond.

Since the calculated molecular weight of the entire EPHA1 cytoplasmic region is 47 kDa, the 60 kDa fragment is predicted to contain not only the intracellular portion of EPHA1 but also the transmembrane helix and part of the extracellular region. Confirming this, the size of the 60 kDa fragment was reduced by PNGase F treatment, indicating that the fragment is N-glycosylated (Fig. 3*F*). Further analysis revealed that the EPHA1 WT and N414A 60 kDa fragments have similar size, whereas the N478A fragment is smaller (Fig. 3*G*). This indicates that the 60 kDa fragment contains the N478 but not the N414 glycosylation site and therefore is likely generated by proteolytic cleavage between the two glycosylation sites (Fig. 3*H*).

**Figure 4:**
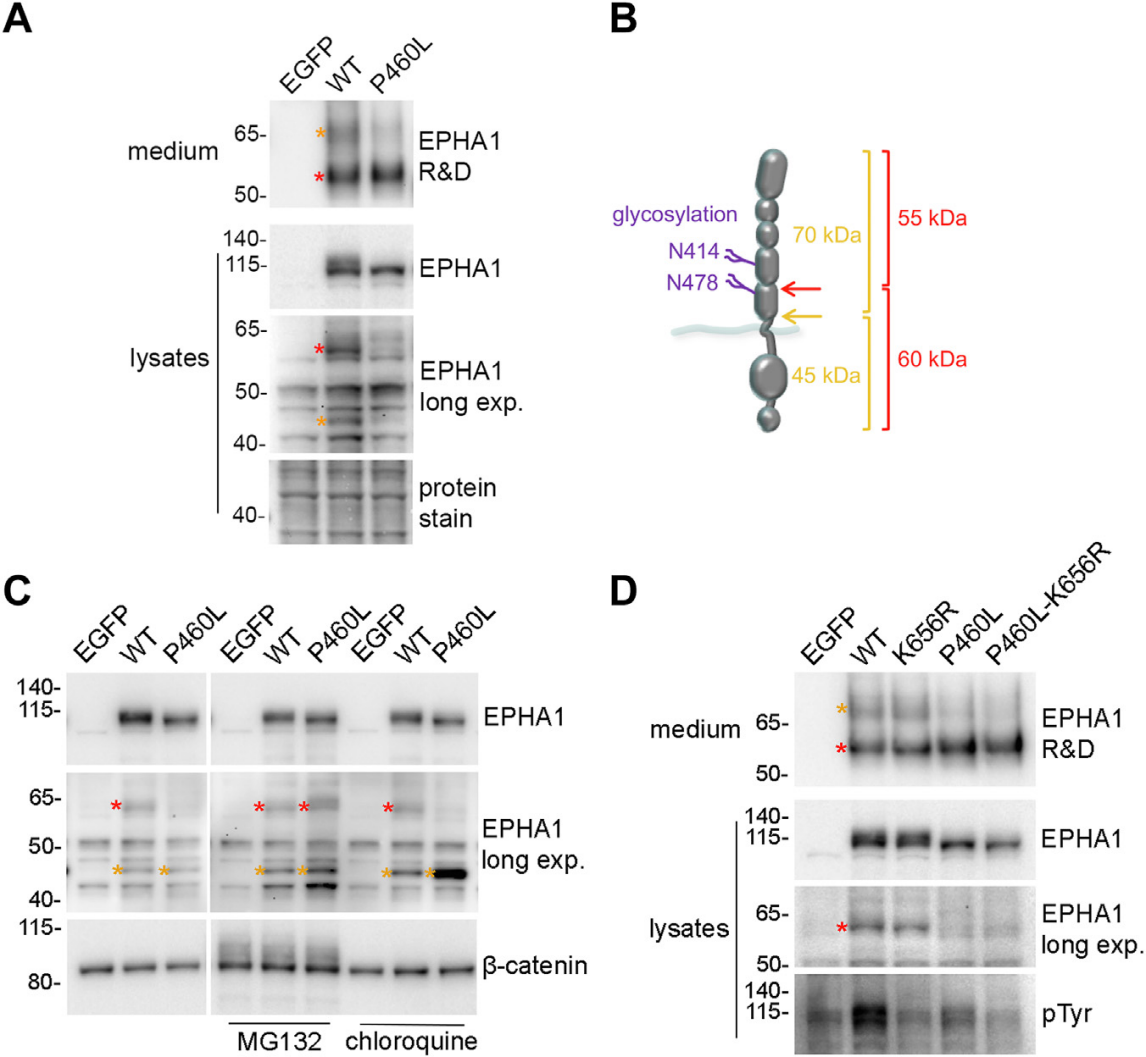
**EPHA1 WT and the P460L mutant are both cleaved in the extracellular region.** HEK293 cells were transiently transfected with constructs encoding EGFP, EPHA1 WT, or the indicated EPHA1 mutants. *A*, EPHA1 extracellular fragments are detected in the culture medium. Conditioned culture medium was probed by immunoblotting with an antibody that recognizes the EPHA1 extracellular region (EPHA1 R&D). Lysates were probed with the EPHA1 SAM antibody and different exposures are shown for the full-length receptor and the less abundant 60 kDa and 45 kDa fragments. Amido black protein stain verifies protein loading. *B*, schematic illustrating two sets of N- and C-terminal fragments generated by two proteolytic cleavages in different parts of the EPHA1 extracellular region. *C*, the P460L cytoplasmic fragment is prone to proteasomal degradation. Cells were treated with MG132 or chloroquine before lysis and probed by immunoblotting with the EPHA1 SAM antibody. The lysates were also probed for β-catenin as a loading control and to verify successful MG132 treatment, as indicated by the nondegraded ubiquitinated β-catenin bands, which have a higher molecular weight. *D*, proteasomal degradation of the 60 kDa fragment does not require EPHA1 kinase activity, since the 60 kDa fragment is degraded even when the P460L mutation is combined with the kinase-inactivating K656R mutation. *Red* asterisks mark the N- and C-terminal fragments generated by EPHA1 cleavage within the FN2 domain; *orange* asterisks mark the N- and C-terminal fragments likely generated by EPHA1 cleavage near the plasma membrane. pTyr, phosphotyrosine.

The proteolytic cleavage should release into the culture medium a corresponding N-terminal fragment of ∼55 kDa (since full-length EPHA1 has an apparent molecular weight of ∼115 kDa). Indeed, an antibody recognizing the EPHA1 extracellular region detects a band of ∼55 kDa in the conditioned cell culture medium of HEK293 cells expressing EPHA1 WT (Fig. 4, *A* and *B*, red). Another fainter band of ∼70 kDa was also detected in the medium, corresponding to a ∼45 kDa fragment recognized by the EPHA1 SAM domain antibody in the cell lysates (Fig. 4, *A* and *B*, orange). The 70 kDa and 45 kDa fragments suggest a second extracellular proteolytic cleavage site located near the plasma membrane.

A 55 kDa fragment was also detected in the conditioned medium of cells expressing EPHA1 P460L, at levels similar to the fragment from EPHA1 WT. This was surprising, given the much lower abundance of the P460L 60 kDa C-terminal fragment presumably generated from the same cleavage event (Fig. 4*A*). To determine whether the P460L 60 kDa fragment might be rapidly degraded, we treated the cells with the proteasome inhibitor MG132 or the lysosome inhibitor chloroquine. Only MG132 increased the abundance of the P460L 60 kDa fragment, consistent with proteasomal degradation (Fig. 4*C*). MG132, and to a greater extent chloroquine, also increased the abundance of the ∼45 kDa fragment derived from EPHA1 P460L, indicating that this smaller fragment is also degraded but mostly in the lysosome. Degradation of the P460L mutant 60 kDa fragment does not depend on EPHA1 kinase activity and tyrosine phosphorylation, since the P460L mutant fragment and the kinase-inactive P460L-K656R double mutant fragment were similarly degraded (Fig. 4*D*).

### Metalloproteases cleave EPHA1 in the second fibronectin type III domain

**Figure 5:**
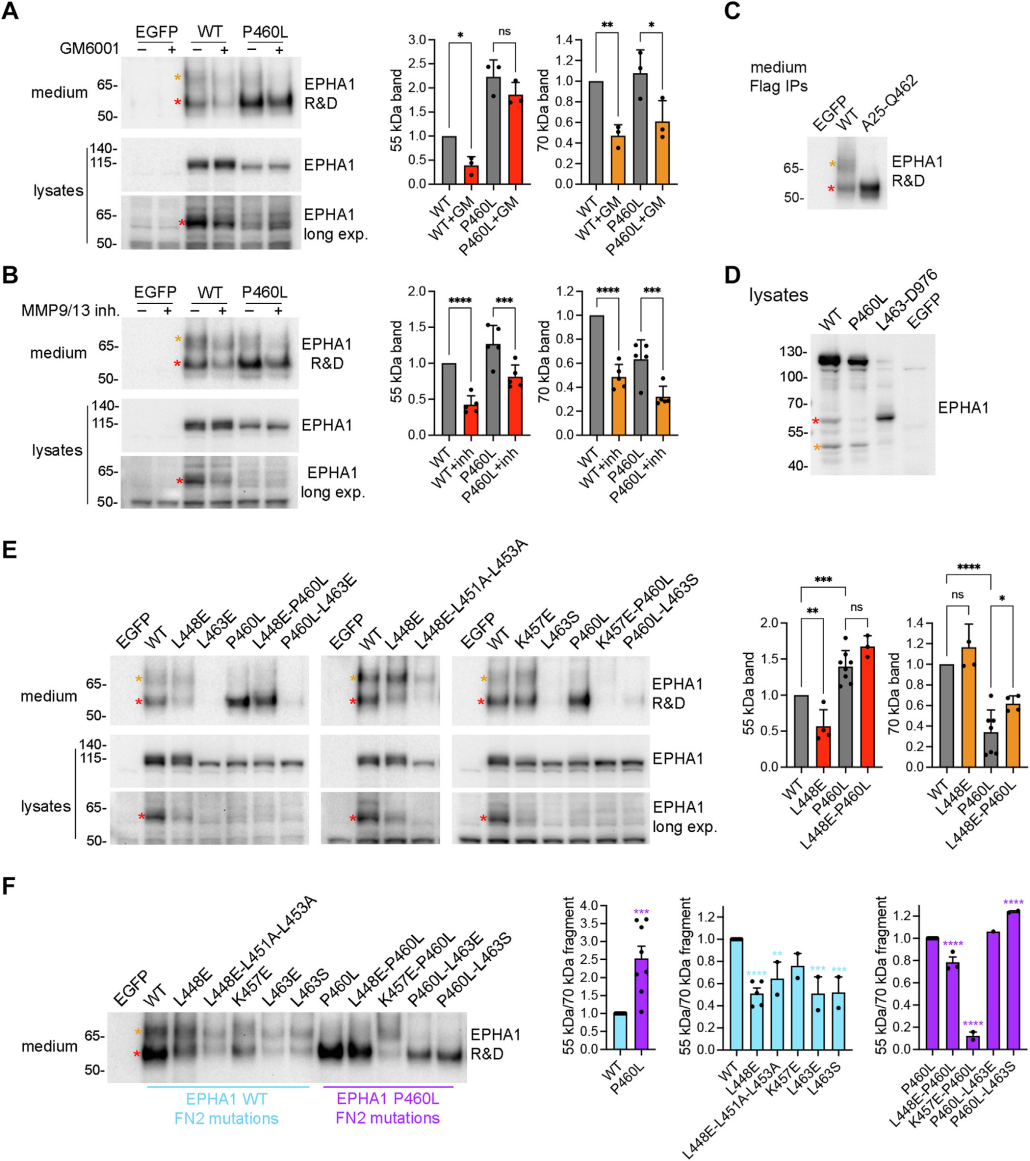
**The EPHA1 ectodomain is cleaved by matrix metalloproteinases.** HEK293 cells were transiently transfected with constructs encoding EGFP, EPHA1 WT, or the indicated EPHA1 mutants. *A* and *B*, after transfection, the cells were incubated without (−) or with (+) the broad-spectrum MMP inhibitor GM6001 (A) or a MMP 9/MMP13 inhibitor (B). Conditioned medium and cell lysates were probed as indicated. *C*, Flag immunoprecipitates from conditioned cell culture medium were probed with an antibody to the EPHA1 extracellular region. EPHA1 A25-Q462 is a Flag-tagged engineered EPHA1 secreted form. *D*, cell lysates were probed with the EPHA1 SAM antibody. L463-D976 is a Flag-tagged engineered EPHA1 truncated form. *E*, conditioned medium and lysates from cells expressing EPHA1 with mutations in predicted FN2 domain MMP proteolytic cleavage motifs were probed as in (A) and (B). *F*, effects of EPHA1 FN2 domain mutations on the relative abundance of the 55 kDa *versus* the 70 kDa fragment in the culture medium. Different amounts of medium were loaded to enable detection of the two bands even when they are present at very low levels. The graphs show means and SEs from the quantification of immunoblots from multiple experiments, with individual data points shown as *black* dots. Three experiments were quantified in both panels in A; five experiments in both panels in B; three to eight (*left* panel) and four to seven (*right* panel) experiments in E; and eight (*left* panel), two to five (*middle* panel), and one to three (*right* panel) experiments in F. ∗, *p* < 0.05; ∗∗, *p* < 0.01; ∗∗∗, *p* < 0.001; and ∗∗∗∗, *p* < 0.0001 for comparisons with WT (*left* and *middle* graphs) or P460L (*right* graph) by one-way ANOVA and Dunnett’s multiple comparison test. *Red* asterisks mark the N- and C-terminal fragments generated by EPHA1 cleavage within the FN2 domain; *orange* asterisks mark the N- and C-terminal fragments generated by EPHA1 cleavage near the plasma membrane.

Eph receptors can be cleaved in the extracellular region by matrix metalloproteinases (MMPs) or a disintegrin and metalloproteinases (ADAMs) (41, 42). Treatment with the general metalloprotease inhibitor GM6001 or with an inhibitor of MMP9 and MMP13 (two MMPs that have been implicated in neurodegeneration (43, 44)) reduced by ≥ 50% the release of both 55 kDa and 70 kDa EPHA1 WT fragments into the culture medium, consistent with the notion that metalloproteases mediate at least in part the cleavages that generate these fragments (Fig. 5, *A* and *B*).

A search for the most likely metalloprotease cleavage sites in the region between N414 and N478 of EPHA1 WT using the ProsperousPlus web resource (45) (prosperousplus.unimelb-biotools.cloud.edu.au) revealed several potential MMP cleavage sites, whereas ADAMs seem less likely to cleave this region (Fig. S4, EPHA1 WT). The top predicted cleavage sites include G427-L428 in the FN1 domain as well as a cluster of four potential cleavage sites (S447-L448, G450-L451, S452-L453, R454-L455) and the Q462-L463 site in the FN2 domain. The S447, L448, G450, L451, and S452 residues are located in a predicted FN2 loop, which may make them more accessible to metalloproteases, while the other residues are located in predicted β-strands (Fig. S2*C*). A construct encoding EPHA1 residues A25-Q462 with a Flag tag generates a secreted protein with a size similar to that of the Flag-tagged 55 kDa N-terminal proteolytic fragment (Fig. 5*C*). Furthermore, a construct encoding EPHA1 residues L463-D976 with a Flag tag generates a protein with a size similar to that of the untagged 60 kDa cell-associated fragment (Fig. 5*D*). Thus, cleavage must occur in the FN2 domain in the vicinity of L463.

Similar to what we observed for EPHA1 WT, the GM6001 and MMP9/MMP13 inhibitors decreased the release of the P460L 70 kDa mutant fragment into the medium (Fig. 5, *A* and *B*). However, these inhibitors were less effective at decreasing generation of the P460L 55 kDa fragment. This suggests that the P460L mutation may facilitate FN2 domain proteolytic cleavage by causing some misfolding that makes potential cleavage motifs in the FN2 domain more accessible to metalloproteases. Increased cleavage of the P460L mutant would also be consistent with the substantial amount of released 55 kDa fragment despite the very low levels of EPHA1 P460L on the cell surface.

We generated the L448E mutant to disrupt the corresponding MMP cleavage motif (Fig. S4). We found that the L448E mutation substantially reduces the amount of both the EPHA1 WT 55 kDa fragment in the medium and the 60 kDa fragment in the cell lysates (Fig. 5*E*, left and middle immunoblots). However, the 55 kDa band was still detectable, suggesting incomplete inhibition of cleavage by the mutation or the presence of additional proteolytic cleavage sites. The L448E mutation did not impair generation of the 70 kDa fragment (Fig. 5*E*), as expected since the cleavage site generating the 70 kDa fragment is likely far from L448. Taken together, these data suggest that S447-L448 is an important proteolytic cleavage site in EPHA1 WT, but not the only one.

The L448E-L451A-L453A triple mutation, designed to inhibit cleavage at two other sites near L448, decreased EPHA1 expression and greatly decreased EPHA1 cell surface localization, as inferred by the loss of the full-length EPHA1 upper band and the extremely low levels of both 55 kDa and 70 kDa fragments in the culture medium (Fig. 5*E*, middle immunoblot). Increased gel loading of the culture medium enabled visualization and quantification of both 55 kDa and 70 kDa fragments even when present at very low levels (Fig. 5*F*). Since the 70 kDa fragment should not be affected by FN2 domain mutations, we normalized the abundance of the 55 kDa band relative to that of the 70 kDa band (Fig. 5*F*, graphs). This confirmed higher generation of the P460L 55 kDa fragment than the corresponding WT fragment (Fig. 5*F*, left graph). In addition, we found that the 55 kDa band is stronger than the 70 kDa band for EPHA1 WT, while it has similar intensity as the 70 kDa band for the L448E mutant, consistent with reduced cleavage of the mutant at the S447-L448 site (Fig. 5*F*). The triple L448E-L451A-L453A mutation did not have greater effect than the L448E mutation alone (Fig. 5*F*, middle graph), suggesting that G450-L451 and S452-L453 are not major cleavage sites. Similar to the triple mutation, mutation of L463 to either glutamic acid or serine to disrupt the predicted cleavage motif also strongly decreased EPHA1 expression, cell surface localization, and cleavage (Fig. 5, *E* and *F*). Both L463E and L463S mutations decreased the 55 kDa band relative to the 70 kDa band, indicating that EPHA1 WT can be cleaved at both the S447-L448 and Q462-L463 sites (Fig. 5*F*, left graph).

Surprisingly, the L448E and L463E/L463S mutations did not substantially decrease the release of the 55 kDa fragment in the case of the EPHA1 P460L mutant (Fig. 5*F*, right graph), consistent with the possibility of an alternative cleavage motif generated by the P460L mutation (Fig. S4, EPHA1 P460 L). Indeed, although the K457E mutation designed to disrupt the E459-L460 cleavage motif introduced by the P460L mutation decreased the already very low surface localization of the P460L mutant (Fig. 5*E*), we observed a strong reduction of the 55 kDa band relative to the 70 kDa band (Fig. 5*F*, right graph). In contrast, the K457E mutation did not significantly affect the cleavage of EPHA1 WT, as expected given the absence of the E459-L460 cleavage motif (Fig. 5*F*, middle panel, and Fig. S4). These results suggest that the EPHA1 P460L mutant is mainly cleaved at the E459-L460 site, which is present only in this mutant.

### EPHA1 Alzheimer’s mutations disrupt receptor phosphorylation

**Figure 6:**
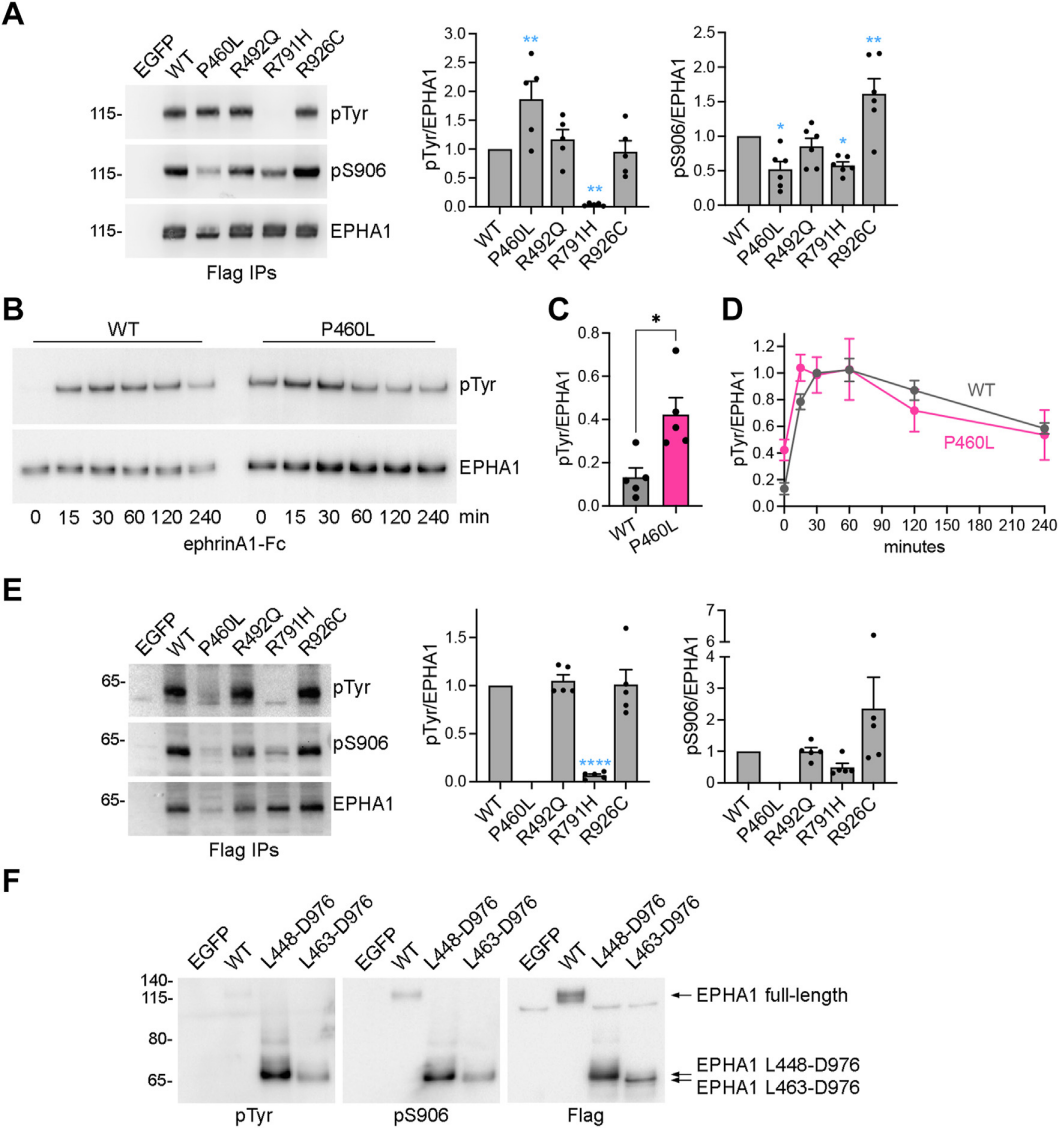
**Some Alzheimer’s mutations disrupt EPHA1 tyrosine and S906 phosphorylation.***A*, Flag immunoprecipitates (IPs) from transiently transfected HEK293 cells were probed by immunoblotting with the indicated antibodies. The graphs show means and SEs for the quantifications of EPHA1 tyrosine phosphorylation (pTyr, n = 5) and S906 phosphorylation (pS906, n = 6) normalized to EPHA1 levels in the upper of the two bands detected with the EPHA1 SAM antibody (which corresponds to the phosphorylated band). Individual data points are shown as *black* dots. ∗, *p* < 0.05 and ∗∗, *p* < 0.01 for the comparison with EPHA1 WT using one-way ANOVA and Dunnett’s multiple comparisons test. *B*, HEK293 cells stably expressing Flag-tagged EPHA1 WT or P460L mutant were stimulated with ephrinA1-Fc for the indicated times and Flag immunoprecipitates were probed by immunoblotting for phosphotyrosine (pTyr) or EPHA1. *C* and *D*, quantifications of the immunoblots in (B) and others (n = 5). The basal EPHA1 tyrosine phosphorylation (pTyr) signal was normalized to EPHA1 levels in the absence of ephrinA1-Fc stimulation and then further normalized to the pTyr/EPHA1 WT signal at 30 min of ephrinA1-Fc stimulation. The graph in (C) shows means and SEs and the individual data points are shown as *black* dots. ∗, *p* < 0.05 by unpaired Student’s *t* test. The graph in (D) shows means ± SEs from five experiments. *E*, experiment similar to A, but using a longer exposure to visualize the EPHA1 60 kDa fragment co-immunoprecipitated with full-length EPHA1 as in Fig. 3, *D* and *E*. The graphs show means and SEs for the quantifications of EPHA1 tyrosine phosphorylation (pTyr, n = 5) and S906 phosphorylation (pS906, n = 5) normalized to EPHA1 levels. The P460L mutant fragment could not be reliably quantified given its very low level. ∗∗∗∗, *p* < 0.0001 for the comparison with WT using one-way ANOVA and Dunnett’s multiple comparisons test. *F*, HEK293 cells were transiently transfected with Flag-tagged EPHA1 WT (comprising residues A25-D976), the Flag-tagged engineered EPHA1 L448-D976 and L463-D976 truncated forms, and EGFP as a control. Immunoblotting for tyrosine phosphorylation (pTyr), S906 phosphorylation, and the Flag tag demonstrates high phosphorylation of the EPHA1 engineered truncated forms.

The signaling ability of EPHA1 depends on phosphorylation on tyrosine residues leading to canonical forward signaling and, presumably, on S906 phosphorylation (phosphosite.org) leading to a noncanonical form of signaling (26) (Figs. S3*B* and 7). To investigate the possible functional effects of the mutations identified in Alzheimer’s patients, we assessed phosphorylation of Flag-tagged EPHA1 in transiently transfected HEK293 cells. After immunoprecipitation with an anti-Flag antibody, substantial tyrosine phosphorylation of EPHA1 WT was detected by immunoblotting with an anti-phosphotyrosine antibody (Fig. 6*A*). Constitutive tyrosine phosphorylation has also been observed for other Eph receptors in transiently transfected HEK293 cells, presumably because the high expression promotes interaction between Eph receptor molecules leading to cross-phosphorylation and kinase activation (46). The tyrosine phosphorylated band corresponds to the upper of the two full-length EPHA1 bands, representing cell surface EPHA1.

**Figure 7:**
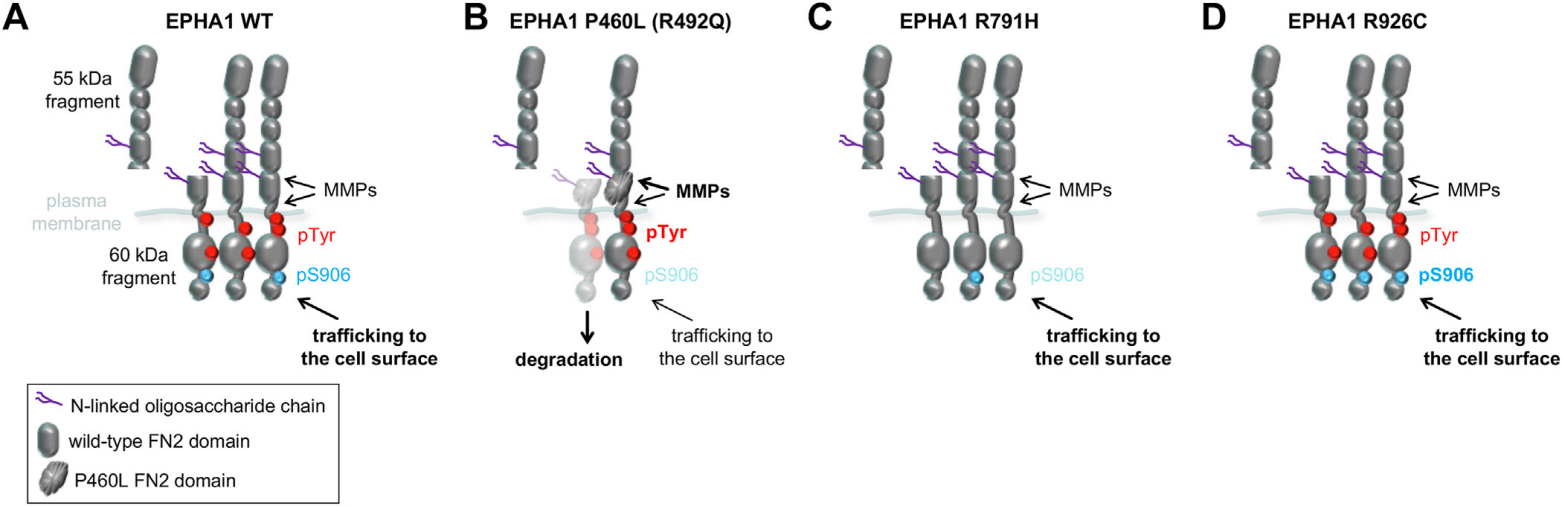
**Schematic illustrating the effects of the EPHA1 Alzheimer’s mutations on EPHA1 signaling features.***A*, EPHA1 WT traffics to the cell surface, where it can signal through tyrosine phosphorylation (pTyr, *red circles*) and S906 phosphorylation (pS906, *blue circle*) and can be cleaved by metalloproteases (MMPs). Cleavage generates truncated fragments released from the cells (a 55 kDa fragment, shown, and a 70 kDa fragment, not shown) or remaining associated with the cells (60 kDa fragment, shown, and 45 kDa fragment, not shown). The 60 kDa cell-associated fragment is phosphorylated on both tyrosine residues and S906, suggesting signaling ability. *B*, the P460L mutation in the FN2 domain decreases EPHA1 levels at the plasma membrane, likely because FN2 domain misfolding impairs EPHA1 trafficking to the cell surface. Full-length EPHA1 P460L on the cell surface is more tyrosine phosphorylated and less phosphorylated on S906 than EPHA1 WT. Matrix metalloproteases cleave the EPHA1 P460L FN2 domain more than EPHA1 WT, but the P460L cell-associated 60 kDa fragment undergoes proteasomal degradation. The R492Q mutation, also in FN2, has effects similar to the P460L mutation but less pronounced. *C*, the R791H kinase domain mutation abolishes kinase activity, and thus autophosphorylation on tyrosine residues, and also decreases S906 phosphorylation. *D*, the R926C mutation in the SAM domain increases S906 phosphorylation. Bold or darker font indicates more pronounced effects. The P460L mutant FN2 domain is shown with a different shape than WT to suggest misfolding.

We found that the P460L mutant is more tyrosine phosphorylated than EPHA1 WT, given the much lower abundance of the mutant upper band but the similar tyrosine phosphorylation signal (Figs. 6, *A* and 7*B*), suggesting that the P460L mutation may increase ligand-independent EPHA1 oligomerization, autophosphorylation, and kinase activity. To examine the effects of the P460L mutation on ligand-induced EPHA1 activation, we used stably transfected HEK293 cells, which have low basal EPHA1 WT tyrosine phosphorylation (Fig. 6*B*). Higher tyrosine phosphorylation of the P460L mutant than WT was apparent in stably transfected HEK293 cells in the absence of ligand stimulation (Fig. 6, *B* and *C*). Treatment with the ephrinA1-Fc ligand increased the tyrosine phosphorylation of both EPHA1 WT and the P460L mutant, with similar maximal tyrosine phosphorylation levels for both WT and mutant observed at 30 to 60 min (Fig. 6, *B* and *D*).

In contrast, the R791H mutant did not show any detectable tyrosine phosphorylation, suggesting that the R791H mutation abrogates kinase activity (Figs. 6*A* and 7*C*). This is perhaps not surprising, since R791 is located in the kinase domain near the activation loop and is conserved in all Eph receptors except the kinase-inactive EPHA10 (31). Modeling suggests that R791 forms several hydrogen bonds with other kinase domain residues (including one hydrogen bond with P826 and two with G828; Fig. S2*D*), implying that the R791H mutation may disrupt the structural integrity of the kinase domain. We also found that the EPHA1 R492Q and R926C mutants exhibit a tyrosine phosphorylation level similar to EPHA1 WT (Fig. 6*A*).

To detect EPHA1 phosphorylation on S906 in the linker between kinase and SAM domains (Fig. S3, *B and C*), we generated a new phosphospecific antibody, since no antibodies recognizing this phosphosite are commercially available. Despite the similarity of the EPHA1 S906 motif with the corresponding EPHA2 S897 motif (Fig. S3*C*), our antibody specifically recognizes EPHA1 phosphorylated on S906 (Fig. S3, *D* and *E*). Conversely, the commercial phosphospecific antibody recognizing the EPHA2 S897 phosphorylation site does not recognize the EPHA1 S906 phosphorylation site (Fig. S3*E*). Similar to the increased phosphorylation of EPHA2 S897 downstream of ERK/RSK and possibly other serine/threonine kinases in HEK293 cells treated with the phorbol ester 12-O-tetradecanoylphorbol-13-a (47), we found that 12-O-tetradecanoylphorbol-13-a treatment also increases EPHA1 S906 phosphorylation (Fig. S3, *D* and *E*). This suggests similar regulation of EPHA1 and EPHA2 noncanonical signaling.

Immunoblotting of EPHA1 immunoprecipitates with the newly developed S906 phosphospecific antibody shows that S906 phosphorylation, like tyrosine phosphorylation, is almost exclusively confined to the EPHA1 upper band corresponding to cell surface EPHA1 (Fig. 6*A*). Furthermore, the P460L and R791H mutations significantly reduced S906 phosphorylation (Figs. 6*A* and 7*B*,C), suggesting that these mutations inhibit EPHA1 noncanonical signaling function. In contrast, the R926C mutation increased EPHA1 S906 phosphorylation (Figs. 6*A* and 7*D*). Modeling suggests that R926 in the SAM domain forms two hydrogen bonds with D824 in the kinase domain (Fig. S2*E*). Thus, the R926C mutation may disrupt interactions between the kinase and SAM domains, possibly making S906 more accessible to serine/threonine kinases.

The WT 60 kDa fragment is also phosphorylated on both tyrosine and S906 (Figs. 6*E* and 7*A*), suggesting that it is capable of signaling. The signaling ability of the 60 kDa fragment seems to be constitutive and not dependent on the presence of full-length EPHA1, since the ∼60 kDa EPHA1 L448-D976 and L463-D976 fragments encoded by plasmids (rather than generated by the cleavage of full-length EPHA1) are also highly phosphorylated on both tyrosine and S906 (Fig. 6*F*). In contrast, the R791H mutant 60 kDa fragment, similar to the corresponding full-length mutant, lacks tyrosine phosphorylation and has greatly reduced S906 phosphorylation (Fig. 6*E*).

## Discussion

Knowledge about the physiological functions of EPHA1 is limited. The new EPHA1 antibodies we developed have enabled us to uncover previously unknown EPHA1 signaling features. We show that EPHA1 phosphorylation on S906, like the corresponding S897 phosphorylation in EPHA2 (48), likely occurs at the plasma membrane (Fig. 7*A*) and is regulated at least in part similarly to EPHA2 S897 phosphorylation (47). This suggests that EPHA1 mediates a noncanonical form of signaling similar to that described for EPHA2 but whose physiological and pathological effects remain to be characterized.

We also uncovered the susceptibility of EPHA1 to proteolytic cleavage by metalloproteases both in the FN2 domain (Fig. 7*A*) and near the transmembrane helix. Although short (3 h) exposure to the general metalloprotease inhibitor GM6001 was previously reported to have small or nonsignificant effects on EPHA1 cleavage (49), we observed effects with longer (2 days) treatment, suggesting slow release of EPHA1 ectodomain fragments that accumulate in the medium over time. Our data suggest that metalloproteases can cleave EPHA1 through a mechanism that is independent of ephrin ligand binding and kinase activity. The metalloproteases predicted as most likely to cleave the FN2 domain of EPHA1 are MMPs, including MMP14 (also known as MT1-MMP), MMP7, and MMP2 (Fig. S4), although the involvement of ADAMs cannot be ruled out (50). EPHA1 extracellular cleavage is a physiological event, since many mass spectrometry studies have detected the EPHA1 ectodomain in human plasma, where it is the most abundant Eph receptor (51) (peptideatlas.org/builds/human/plasma/). Other Eph receptors can also be cleaved by MMPs (41, 42). For example, a cell-associated EPHA2 ∼60 kDa fragment generated by MMP14-mediated cleavage in the FN1 domain has been implicated in breast cancer cell invasiveness through S897 phosphorylation (52). MMPs can play diverse roles in neuroinflammation and neurodegeneration (43, 44, 53), but the importance of MMP-mediated EPHA1 cleavage in Alzheimer’s disease remains to be determined.

EPHA1 cleavage could have functional consequences on the signaling activity of both EPHA1 and ephrinAs. First, by generating a truncated form of EPHA1 lacking the ligand-binding domain, cleavage decreases ephrinA-induced EPHA1 forward signaling as well as EPHA1-induced ephrinA reverse signaling (26). Second, the N- and C-terminal proteolytic fragments generated by EPHA1 cleavage could have signaling roles. The transmembrane 60 kDa fragment can be phosphorylated on both tyrosine residues and S906, suggesting its ability to mediate both kinase-dependent signaling and noncanonical signaling. The shed EPHA1 fragments, which contain the ligand-binding domain, could bind ephrinA ligands and prevent their interaction with full-length EphA receptors, thus presumably inhibiting both EphA forward signaling and ephrinA reverse signaling (42). Concentrations of the EPHA1 extracellular fragment much higher than those detected in plasma (estimated to be ∼20 ng/ml, corresponding to ∼0.3 nM) (51) (peptideatlas.org/builds/human/plasma/) would likely be required for these effects (49). Such concentrations may be achieved locally within tissues near cells with high EPHA1 expression. The EPHA1 ectodomain in the blood might also serve as a useful disease biomarker, if EPHA1 ectodomain shedding is perturbed by pathological processes.

A role for EPHA1 in late-onset Alzheimer’s disease was first proposed almost 15 years ago, but the involvement of this receptor in disease pathogenesis is still poorly understood. Our data show that the P460L mutation identified in several Alzheimer’s disease families disrupts EPHA1 function through multiple mechanisms (Fig. 7*B*). The most prominent effect we uncovered is a marked decrease in the cell surface localization of the P460L mutant, as indicated by the low abundance of the full-length EPHA1 upper band relative to the lower band. The separation of EPHA1 into two closely spaced bands in immunoblots has not been previously reported, possibly because the two bands are not clearly distinguishable in some commonly used types of SDS-PAGE gels (our unpublished observation). In agreement with our finding that the lower band corresponding to intracellular EPHA1 is predominant in the case of the P460L mutant, recent flow cytometry and immunofluorescence data have shown preferential EPHA1 P460L localization in intracellular compartments (49).

In a structural model of EPHA1, the P460 residue is in a predicted loop (Fig. S4, *A* and *B*). Therefore, its mutation to leucine may affect the conformation of the loop, with potential consequences on FN2 domain folding. We propose that the low cell surface levels of EPHA1 P460L are likely due to impaired trafficking to the cell surface (Fig. 6) rather than higher proteolytic cleavage or degradation, since only a small fraction of the EPHA1 molecules appear to be proteolytically cleaved (Fig. 3, *A* and *C*) and the K457E mutation almost completely inhibits cleavage but does not detectably increase the upper band in the EPHA1 K457E-P460L mutant (Fig. 5, *E* and *F*). Preventing proteasomal or lysosomal degradation also did not detectably increase the full-length P460L upper band representing the cell surface–localized receptor (Fig. 4*C*). Consistent with the hypothesis that the P460L mutation impairs trafficking to the plasma membrane (Fig. 7), the mutation also decreased secretion of an engineered EPHA1 fragment comprising the entire ectodomain (not shown).

Our data highlight the FN2 domain as a particularly important region of EPHA1 that is highly sensitive to amino acid changes. We found that multiple amino acid mutations in the FN2 domain can dramatically reduce the abundance of the EPHA1 upper band, suggesting impairment of receptor trafficking to the cell surface. Interestingly, half of the eight identified Alzheimer’s missense mutations are in the FN2 domain. This domain also contains a cancer “3D clustered” mutation hotspot that includes V514 (cbioportal.org), a residue also found to be mutated in an Alzheimer’s patient (Fig. S1 and Table S1) and not yet characterized.

Since our evidence suggests that cell surface EPHA1 is the main phosphorylated species, reduced levels of full-length EPHA1 P460L on the cell surface should result in decreased abundance of tyrosine phosphorylated receptor. However, we detected a higher tyrosine phosphorylation signal for the P460L mutant than for EPHA1 WT in both transiently and stably transfected HEK293 cells, indicating that the P460L mutation increases ligand-independent EPHA1 tyrosine phosphorylation (Fig. 7*B*). An earlier study also detected increased tyrosine phosphorylation of the P460L mutant compared to WT in transfected HEK293 cells (40). The authors proposed that the higher hydrophobicity of L460 than P460 promotes interaction of the EPHA1 FN2 domain with plasma membrane lipids, increasing receptor oligomerization, tyrosine phosphorylation, and downstream signaling involving RAC1, RHOA, and ERK (40). Our findings also reveal that the P460L mutant remains responsive to ligand stimulation. A 30 or 60 min stimulation with ephrinA1-Fc induced similar maximal tyrosine phosphorylation of EPHA1 WT and P460L, although starting from higher basal phosphorylation for the P460L mutant. In contrast, another recent study using stably transfected HEK293 cells and measuring EPHA1 phosphorylation on Y781 in the activation loop reported lack of responsiveness of EPHA1 P460L to 5 min ephrinA1-Fc stimulation (49), perhaps due to the short stimulation time. Alternatively, the P460L mutant may exhibit a slower response to ephrinA1-Fc than EPHA1 WT or the P460L mutation may preferentially impair phosphorylation on Y781.

We also found that the P460L mutation substantially increases metalloprotease-mediated EPHA1 cleavage. Our data show that the 55 kDa ectodomain fragment released by proteolytic cleavage of the P460L mutant is more abundant than the EPHA1 WT fragment (Figs. 5*F* and 7, *A* and *B*), even though full-length EPHA1 P460L levels on the cell surface—where the cleavage presumably occurs—are very low. This may be due to the new MMP cleavage site introduced by the P460L mutation between E459 and L460. The P460L mutation might in addition facilitate cleavage by causing FN2 domain misfolding and/or by promoting EPHA1 interaction with a transmembrane MMP such as MMP14, which is known to interact with the closely related EPHA2 receptor (52). Interestingly, the P460L mutation might synergize with the higher MMP activity associated with Alzheimer’s disease (43, 44, 53) to increase EPHA1 proteolysis.

A recent study also reported EPHA1 ectodomain cleavage but less pronounced for the P460L mutant than for EPHA1 WT, although it is not clear whether the ELISA kit used in that study might preferentially detect the 70 kDa fragment over the 55 kDa fragment (49). The authors also reported that pretreatment with high (1000–5000 ng/ml) concentrations of the entire EPHA1 ectodomain dimerized by fusion to Fc activates ephrinA reverse signaling in cultured brain endothelial cells, promoting their ability to interact with immune cells. Furthermore, the WT ectodomain Fc fusion was reported to increase blood-brain barrier permeability, which could facilitate immune cell penetration into the brain. These effects were not observed with the P460L ectodomain Fc fusion. However, it is not known whether the fragments released from endogenous EPHA1 also exhibit this activity. In addition, the more abundant 55 kDa fragment does not contain P460 and thus does not differ between EPHA1 WT and the P460L mutant.

Another effect of the P460L mutation that we uncovered is a drastic decrease in the abundance of the EPHA1 60 kDa C-terminal proteolytic fragment due to proteasomal degradation (Fig. 7*B*). Although the P460L mutation also increases EPHA1 tyrosine phosphorylation, this does not seem to be responsible for the degradation of the 60 kDa fragment, since the EPHA1 kinase-inactive P460L mutant undergoes similar degradation. This is different from what has been reported for EPHA2, which undergoes tyrosine phosphorylation-induced proteasomal degradation (54). In the case of EPHA1, the P460L mutation may destabilize the 60 kDa fragment leading to its degradation, similar to what has been reported for missense mutations in the closely related EPHA2 receptor (55).

The R492Q, R791H, and R926C mutations were not previously investigated. We found that the R492Q mutation has effects similar to the P460L mutation but less pronounced (Fig. 7*B*). We also found that the R791H mutation in the kinase domain has especially drastic loss-of-function effects because it abrogates EPHA1 kinase-dependent signaling in both EPHA1 full-length and the 60 kDa fragment (Fig. 7*C*). It also strongly impairs S906 phosphorylation. Finally, the R926C mutation in the SAM domain potentiates S906 phosphorylation without significant effects on kinase-dependent signaling (Fig. 7*D*). It remains to be determined whether dysregulated EPHA1 noncanonical signaling has neuropathological effects.

Our data show that all four Alzheimer’s mutations we have characterized disrupt EPHA1 physiological signaling, but the specific effects depend on the particular mutation. Both gain-of-function (40) and loss-of-function (49) effects of the P460L mutation have been proposed to play a role in Alzheimer’s disease. The effects we observed for P460L and the other mutations examined are consistent with the idea that both gain and loss of EPHA1 function can have pathological effects. It should also be noted that the mutations are likely heterozygous and thus only affect a portion of the expressed EPHA1 receptor. Nevertheless, the mutant EPHA1 may oligomerize with co-expressed WT receptor affecting its signaling output. For example, the R791H mutant might decrease tyrosine phosphorylation of co-expressed EPHA1 WT (56, 57).

More work is needed to elucidate the physiological role of the different EPHA1 signaling features and how their disruption may lead to neurodegeneration. A difficulty hindering further investigation of the role of EPHA1 in Alzheimer’s disease is that the cell type(s) affected by EPHA1 signaling are unknown. A study in *Drosophila* uncovered effects of targeted neuronal misexpression of EPHA1 on arousal, sleep patterns, and circadian behavior but with no differences between EPHA1 WT and the P460L mutant (58). Another study linked increased EPHA1 expression to increased neuroinflammation in a neuronal SH-SY5Y cell culture model of Parkinson’s disease as well as in the substantia nigra of a mouse Parkinson’s disease model, but the effects of EPHA1 mutations were not examined (59). EPHA1 is expressed at very low levels in the nervous system (60, 61) (proteinatlas.org) and has not been detected in cerebrospinal fluid despite being easily detectable in human plasma (peptideatlas.org). In contrast, EPHA1 is highly expressed in undifferentiated embryonic and induced pluripotent stem cells (62, 63, 64), epithelial cells (20, 65), and the immune system (66, 67, 68, 69). Given the importance of inflammatory processes in the pathology of Alzheimer’s disease (70) and of the immune system in the clearance of Aβ oligomers (71), a role of EPHA1 in immune cells seems the most likely (49, 72, 73). However, experimental evidence supporting this role remains limited (74).

Other EPH receptors have also been implicated in Alzheimer’s disease pathogenesis. For example, EPHA4, EPHB2, and EPHB6 are highly expressed in the brain and their ectodomains are readily detected in cerebrospinal fluid (peptideatlas.org). EPHB6 levels in cerebrospinal fluid were found to correlate with the levels of Tau and phosphorylated Tau, two biomarkers of Alzheimer’s disease pathology (75). Increased EPHA4 signaling but decreased EPHB2 signaling have been reported to exacerbate Alzheimer’s disease pathology in mouse models (76, 77, 78, 79, 80, 81). In addition, altered alternatively splicing of the *EPHB6* gene (located on chromosome 7q34 in the vicinity of the *EPHA1* gene) has been associated with Alzheimer’s disease risk (82) and a rare autosomal *EPHA6* copy number gain was found to co-segregate with familial Alzheimer’s disease status (83). Here we report the functional consequences of EPHA1 missense mutations identified in Alzheimer’s disease patients, including impaired EPHA1 trafficking, decreased protein stability, and dysregulated canonical and/or noncanonical signaling (Fig. 7). Each mutation has distinctive effects that suggest that either decreased or increased EPHA1 signaling may contribute to neurodegeneration, underscoring the physiological importance of balanced EPHA1 activity for nervous system homeostasis.

## Experimental procedures

### Constructs

A cDNA encoding human EPHA1 residues A25-D976 (GenBank accession number BC130291) was cloned between the NotI and BglII sites of pFLAG-CMV3 (E6783, Sigma), in frame with the preprotrypsin leader and the Flag tag to generate the pFLAG-CMV3-EPHA1 construct encoding full-length EPHA1 with an N-terminal Flag tag. The EPHA1 cDNA was excised from pFLAG-CMV3-EPHA1 by digestion with NotI and BglII and subcloned between the NotI and BamHI sites of the pLVX-IRES-Neo vector (632181, Takara Bio) engineered to also encode the preprotrypsin leader and the Flag tag from pFLAG-CMV3, yielding pLVX-Flag-EPHA1 WT.

pFLAG-CMV3-EPHA1 was used as a template to generate the mutants identified in Alzheimer’s patients (P460 L, R492Q, R791H, R926C) by site-directed mutagenesis using the QuickChange method. Mutated fragments were then subcloned to replace the corresponding portion of pLVX-Flag-EPHA1 WT (NotI-BglII fragment for P460L, NotI-XbaI fragment for R492Q, and XbaI-EcoRI fragments for R791H and R926C).

pLVX-Flag-EPHA1 WT was used as a template to generate all other EPHA1 mutant constructs, including N-glycosylation–deficient mutants (N414A, N478A, N414A-N478A, N414A-P460L, P460L-N478A, N414A-P460L-N478A), mutants lacking predicted MMP cleavage sites (L448E, L448E-P460L, L448E-L451A-L453A, K457E, K457E-P460L, L463E, P460L-L463E, L463S, P460L-L463S), truncated mutants (A25-Q462, A25-E547, L463-D976, L448-D976), and a mutant defective in S906 phosphorylation (S906A). All of these mutants were generated by overlapping PCR or, in the case of the truncated mutants, by PCR with primers containing restriction sites appropriate for subcloning.

cDNAs encoding the human EPHA1 kinase-SAM linker and SAM domain (residues T892-D976) and the human EPHA2 kinase-SAM linker and SAM domain (residues T883-I976) were amplified by PCR and subcloned in the NdeI and XhoI sites of the pET-28a vector (69864-3, Novagen). This yielded constructs encoding the MGSSHHHHHHSSGLVPRGSHM sequence, including an N-terminal His tag, followed by the EPHA1 or EPHA2 sequences with stop codons at the end. All amplified regions used to generate constructs were verified by sequencing.

### Antibodies and WGA

The human EPHA1 R&D goat polyclonal antibody (AF638, R&D Systems/BioTechne; 1:1000 dilution for immunoblotting) was obtained using the entire EPHA1 ectodomain as the antigen. Other commercial antibodies used for immunoblotting include antibodies recognizing EPHA2 (6997, Cell Signaling Technology; 1:1000 dilution), IGF1Rβ (3027S, Cell Signaling Technology; 1:1000 dilution), GAPDH (2118S, Cell Signaling Technology; 1:8000 dilution), β-tubulin (2128S, Cell Signaling Technology; 1:1000 dilution), Flag (F1804, Sigma-Aldrich; 1:1000 dilution), β-catenin (9562S, Cell Signaling Technology; 1:1000 dilution), the EPHA2 S897 phosphorylated motif (6347S, Cell Signaling Technology; 1:1000 dilution), and phosphotyrosine (P-Tyr-100 HRP conjugate, 5465S, Cell Signaling Technology; 1:2000 dilution). The secondary antibodies conjugated to horseradish peroxidase used for immunoblotting include goat anti-rabbit (A16110, Life Technologies; 1:4000 dilution), donkey anti-goat (A16005, Life Technologies; 1:4000 dilution), and goat anti-mouse (A16078, Life Technologies; 1:4000 dilution). WGA coupled to horseradish peroxidase (29073, Biotium; 1:1000 dilution) was used to detect EPHA1 oligosaccharide chains containing sialic acid.

To generate the EPHA1 SAM antibody (used to detect full-length EPHA1 and the ∼60 kDa C-terminal fragment), we transformed BL21(DE3)T1R *Escherichia coli* (B2935; Sigma-Aldrich) with the pET-28 constructs encoding kinase-SAM linker and SAM domain of EPHA1 and EPHA2. The expressed His-tagged EPHA1 and EPHA2 proteins were purified by nickel affinity chromatography and the EPHA1 protein was used to immunize rabbits. The immune serum was affinity purified using the His-tagged EPHA1 protein coupled to an Affi-Gel 10 column (1536046; Bio-Rad Laboratories). The purified antibody was then cross-absorbed on a column with the closely related EPHA2 protein to eliminate antibodies recognizing epitopes conserved in other Eph receptors (Fig. S3*A*). The purified antibody was used for immunoblotting at a concentration of 0.15 μg/ml.

To generate an antibody recognizing EPHA1 phosphorylated on S906, the peptide KMTLRLPpSLSGSDG corresponding to EPHA1 residues 900 to 912 with phosphorylated S906 and an added N-terminal lysine, was coupled to bovine serum albumin using glutaraldehyde and used to immunize rabbits. Antibodies recognizing the nonphosphorylated S906 motif were then removed from the immune serum by affinity purification using the nonphosphorylated peptide (KMTLRLPSLSGSDG) coupled to an Affi-Gel 10 column. The absorbed serum was then affinity purified using the phosphorylated peptide (KMTLRLPpSLSGSDG) coupled to an Affi-Gel 10 column. The purified antibody was used for immunoblotting at a concentration of 0.15 μg/ml. The antibody specifically recognizes EPHA1 phosphorylated on S906, since it labels EPHA1 WT overexpressed by transient transfection in HEK293 cells but not the EPHA1 S906A mutant, which lacks the S906 phosphorylation site (Fig. S3*D*). The EPHA1 S906 phosphospecific antibody also does not recognize EPHA2 phosphorylated on S897, a residue that is part of a sequence motif closely related to the S906 motif (Fig. S3, *C* and *E*).

### Cell culture and transfection

HEK293AD human embryonic cells (AD-100, Cell Biolabs) were cultured in Dulbecco's Modified Eagle Medium (#10-013-CV, Corning) containing 10% fetal bovine serum and 1% antimycotics and antibiotics (#30-004-Cl, Corning). For immunoblotting, cell surface biotinylation, and immunoprecipitations, HEK293AD cells were transiently or stably transfected using Lipofectamine 2000 reagent (#11668019, Invitrogen, Thermo Fisher Scientific) according to the manufacturer’s recommendations. A total of 270 ng of plasmid DNA was used for transfections of wells from a 12-well plate, with the amount of each EPHA1 plasmid adjusted between 160 ng and 230 ng to obtain similar expression levels from different EPHA1 constructs and empty vector DNA added to achieve the same total plasmid DNA in all transfections. Twenty-four to forty-eight hours after transfection, the cells were used for experiments or treated with 1 mg/ml G418 (Thermo Fisher Scientific 10131035) for 11 to 15 days to generate stably transfected cells. Cell culture medium (either Dulbecco's Modified Eagle Medium with serum or Opti-MEM without serum) conditioned by the cells for 48 to 72 h was collected for immunoprecipitation or immunoblotting. For ligand stimulation, cells were treated with ephrinA1-Fc (602-A1-200, R&D Systems/BioTechne; 2 μg/ml) for different time periods. To measure the half-life of EPHA1 WT or the P460L mutant, stably transfected HEK293AD cells were treated with cycloheximide (C7698, Sigma-Aldrich; 50 μg/ml) and collected after different time periods. To inhibit EPHA1 proteasomal degradation, cells were treated with MG132 (NC9819784, Thermo Fisher Scientific; 5 μg/ml) for 8 to 9 h. To inhibit EPHA1 lysosomal degradation, cells were treated with chloroquine (ICN19391910, Thermo Fisher Scientific; 10 μg/ml) for 8 to 9 h. To inhibit metalloproteases, cells were treated with GM6001 (NC9924174, Thermo Fisher Scientific; 25 μM) or MMP9/MMP13 inhibitor (CAS 204140-01-2, 444,252, Sigma-Aldrich; 10 μM) for 2 days, with addition of the inhibitor at day 0 and then again at day 1.

### Immunoprecipitation

For immunoprecipitations, cells were cultured until they reached ∼80% confluency and lysed. Alternatively, for ephrinA1-Fc stimulation, cells were rinsed with prewarmed PBS containing calcium and magnesium and serum-starved for 1 h, incubated for 10 min at 37 °C with 2 μg/ml ephrinA1-Fc (602-A1, R&D Systems,) or human Fc (55911, MP Biomedicals) as a control, and then washed with ice-cold PBS containing calcium and magnesium. Cells were lysed in 0.5% TX-100 in PBS containing Halt Protease and Phosphatase inhibitor cocktail (784422, Thermo Fisher Scientific) by incubation for 5 min on ice with periodic mixing. Cell lysates were centrifuged at 4 °C for 10 min at 16,700 g to remove insoluble material. The supernatant was further precleared by incubation with Sepharose beads for 15 min at 4 °C on a rotator followed by centrifugation. Each Flag immunoprecipitation was performed by incubating 20 to 25 μl of anti-Flag M2 affinity gel (A2220, Sigma-Aldrich) with cell lysates for 2 h at 4 °C on a rotator. The immunoprecipitates were washed three times with 1 ml PBS with 0.5% TX-100 and once with PBS and eluted by incubation at 95 °C for 2 min in 25 μl Bolt LDS-containing sample buffer without β-mercaptoethanol (to avoid dissociating the chains of the Flag antibody not covalently linked to the beads). Following centrifugation for 1 min at 1000 g, the supernatant was collected and β-mercaptoethanol was added to a final concentration of 2.5%.

To disrupt the association of full-length EPHA1 with the 60 kDa fragment before immunoprecipitation with Flag antibody, cells were lysed in modified PBS-RIPA buffer (1% Triton X-100, 0.5% sodium deoxycholate, 1% SDS, and 2 mM EDTA in PBS, pH 7.5) with Halt Protease and Phosphatase inhibitor cocktail. Alternatively, cells were lysed in a small volume of modified PBS-RIPA buffer containing 1% SDS instead of the usual 0.1% SDS and incubated in the lysis buffer for 5 min on ice with periodic mixing, before heating the resulting lysates at 95 °C for 2 min followed by 10-fold dilution with modified PBS-RIPA buffer without SDS and immunoprecipitation.

### Immunoblotting and WGA labeling

To obtain cell lysates for immunoblotting, stable cell lines or transiently transfected cells were rinsed once with ice-cold PBS containing Ca+ and Mg+ (17-513F, Lonza) and collected in SDS sample buffer or Bolt LDS sample buffer (B0007, Life Technologies) with 2.5% β-mercaptoethanol. Lysates were heated at 95 °C for 2 min, briefly sonicated, and similar amounts of protein were loaded for each sample, as verified by amido black protein stain (not shown). For immunoblotting of conditioned culture medium, the medium (OptiMEM without serum) incubated with cells for 2 to 3 days was centrifuged for 1 min at 1000 g to remove cell debris, and LDS sample buffer and β-mercaptoethanol to a final concentration of 2.5% were added before heating at 95 °C for 2 min.

Lysates, culture medium, and immunoprecipitates were separated on Bolt 4 to 12% Bis-Tris Plus polyacrylamide gels (NW04125Box, Thermo Fisher Scientific) using MOPS SDS running buffer (B000102, Thermo Fisher Scientific) or 3 to 8% NuPAGE Tris-Acetate gels (EA0375BOX, Thermo Fisher Scientific) using Tris-Acetate Running Buffer(LA0041, Life Technologies). After wet or semi-dry transfer, Immobilon membranes were blocked with 5% bovine serum albumin in 0.1% Tween 20 in TBS (150 mM NaCl, 50 mM Tris–HCl, pH 7.5) for 1 h and then incubated in the cold overnight with primary antibodies. After washing, the membranes were incubated with a horseradish peroxidase–conjugated anti-rabbit secondary antibody (A16110, Invitrogen;1:4000 dilution). The chemiluminescence signal was captured using the ChemiDoc Touch Imaging System (Bio-Rad), quantified using Image Lab (Bio-Rad), and analyzed using Prism software (GraphPad).

### Deglycosylation

Deglycosylation experiments were performed using HEK293 cells transiently transfected with EPHA1 WT or P460L mutant and the PNGase F Glycan Cleavage Kit (A39245, Thermo Fisher Scientific). Cells solubilized in lysis buffer (0.5% TX-100 in PBS, containing Halt Protease and Phosphatase inhibitor cocktail), followed by centrifugation at 16,000 g for 10 min at 4 °C. Supernatants were recovered, and protein quantification was performed with the Pierce BCA Protein Assay Kit (PI23227, Thermo Fisher Scientific). Each lysate was subdivided into three tubes. One tube was immediately denatured by incubation with Bolt LDS sample buffer for 5 min at 95 °C and snap frozen in dry ice. The second tube was diluted in PNGase F reaction buffer without PNGase F enzyme and incubated for 1 h at 50 °C. The third tube was diluted in PNGase F reaction buffer with PNGase F enzyme and incubated for 1 h at 50 °C, according to the manufacturer’s protocol. After the PNGase F enzyme reaction, the samples were denatured by incubation with Bolt LDS sample buffer for 5 min at 95 °C and snap frozen in dry ice.

### Cell surface biotinylation

Cells were washed three times with ice-cold PBS and then incubated with 0.6 mg/ml EZ-Link Sulfo-NHS-SS-Biotin (PI21331, Pierce/Thermo Fisher Scientific) for 30 min on ice before three washes with 100 mM glycine in PBS to quench free reactive biotin and one wash with PBS. Cells were lysed with PBS-RIPA lysis buffer (1% TX-100, 0.5% sodium deoxycholate, 0.1% SDS in PBS, pH 8) supplemented with Halt protease and phosphatase inhibitors (#78442, Thermo Fisher Scientific). Lysates were incubated for 15 min on ice and centrifuged at 16,000 g for 10 min at 4 °C. The protein concentration of the resulting supernatants was determined using a BCA kit (#23225, Pierce/Thermo Fisher Scientific). A portion of the lysate was kept as a whole input reference sample (lysate). Biotinylated proteins were purified by pull down on streptavidin beads (#20327, Thermo Fisher Scientific) for 2 h to overnight at 4 °C on a shaker. Beads were subsequently washed three times with lysis buffer, followed by one PBS wash, and biotinylated proteins were eluted in 2 × LDS buffer supplemented with 2.5% β-mercaptoethanol and heated for 2 min at 95 °C. Total and surface EPHA1 levels were determined by immunoblotting with the homemade antibody recognizing the EPHA1 SAM domain. The blots were also probed with an anti-IGF1Rβ antibody (#3027, Cell Signaling Technology) as a transmembrane protein control and with an anti-GAPDH antibody (#2118S, Cell Signaling Technology) as a cytoplasmic protein control.

## Data availability

All data are contained within the manuscript.

## Supporting information

This article contains supporting information (13, 14, 15, 16).

## Conflicts of interest

The authors declare that they have no conflicts of interest with the contents of this article.
