# Supplementary material for: Missense mutations of the ephrin receptor EPHA1 associated with Alzheimer’s disease disrupt receptor signaling functions
Source: J Biol Chem. 2024 Dec 18;301(2):108099. doi: 10.1016/j.jbc.2024.108099 (PMC11773478; doi:10.1016/j.jbc.2024.108099)
Supplement: Supplementary Figures and Table [file mmc1.pdf]

SUPPORTING INFORMATION

Missense Mutations of the Ephrin Receptor EPHA1 Associated with Alzheimer's Disease Disrupt Receptor Signaling Functions

Mike Matsumoto[1#], Maricel Gomez-Soler[1#], Sara Lombardi[1],
Bernhard C. Lechtenberg[1] and Elena B. Pasquale[1*]

The Supporting information includes Table S1 and Figures S1 to S4.

Table S1. EphA1 missense mutations identified in Alzheimer's patients							
**rsID***	**Mutation**	**MAF****	**reference > alternative allele**	**EPHA1 domain**	**Polyphen prediction**	**Number of patients with the mutation**	**references**
rs201581948	R337Q	0.0002818	C>T	FNIII-1	benign	2	13
rs202178565	P460L	0.0002192	G>A	FNIII-2	probably damaging	≥6 (in 3 cohorts)	13
rs143535859	R471Q	0.002582	C>T	FNIII-2	benign	not reported	13
rs11768549	R492Q	0.01227	C>T	FNIII-2	benign	4 (in 3 studies)	14-16
rs567483385	V514I	0.00009547	C>T	FNIII-2	benign	1	13
rs139711610	R791H	0.0003256	C>T	kinase	probably damaging	3	13
rs537988053	H888Y	0.00001592	G>A	kinase	benign	1	13
rs138715519	R926C	0.0001099	G>A	SAM	probably damaging	1	13

*rsID, reference SNP cluster ID; ** MAF, minor allele frequency from gnomAD.

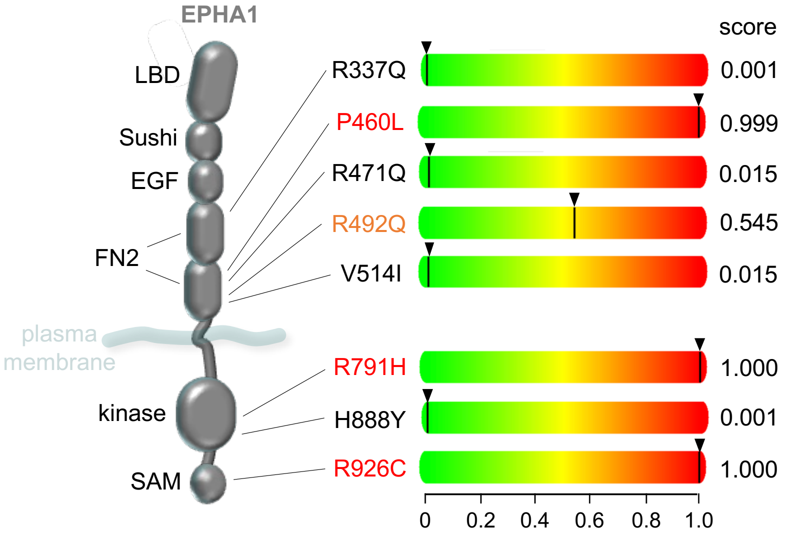

Figure S1. EPHA1 missense mutations identified in Alzheimer's patients. (**Left**) Schematic representation of the EPHA1 domain structure. Abbreviations: LBD, ligand-binding domain; EGF, EGF-like domain; FN2, fibronectin type III domain; SAM, sterile alpha motif domain. (**Right**) PolyPhen-2 predictions of the functional effects of the mutations (http://genetics.bwh.harvard.edu/pph2). The scores of 0.999 and 1.00 indicate mutations that are highly likely to affect the functional properties of the receptor (the names of these mutations are in red font), the score of 0.545 indicates a mutation that may affect functional properties (in orange font), and the scores close to 0 indicate mutations unlikely to have a functional effect (black font).

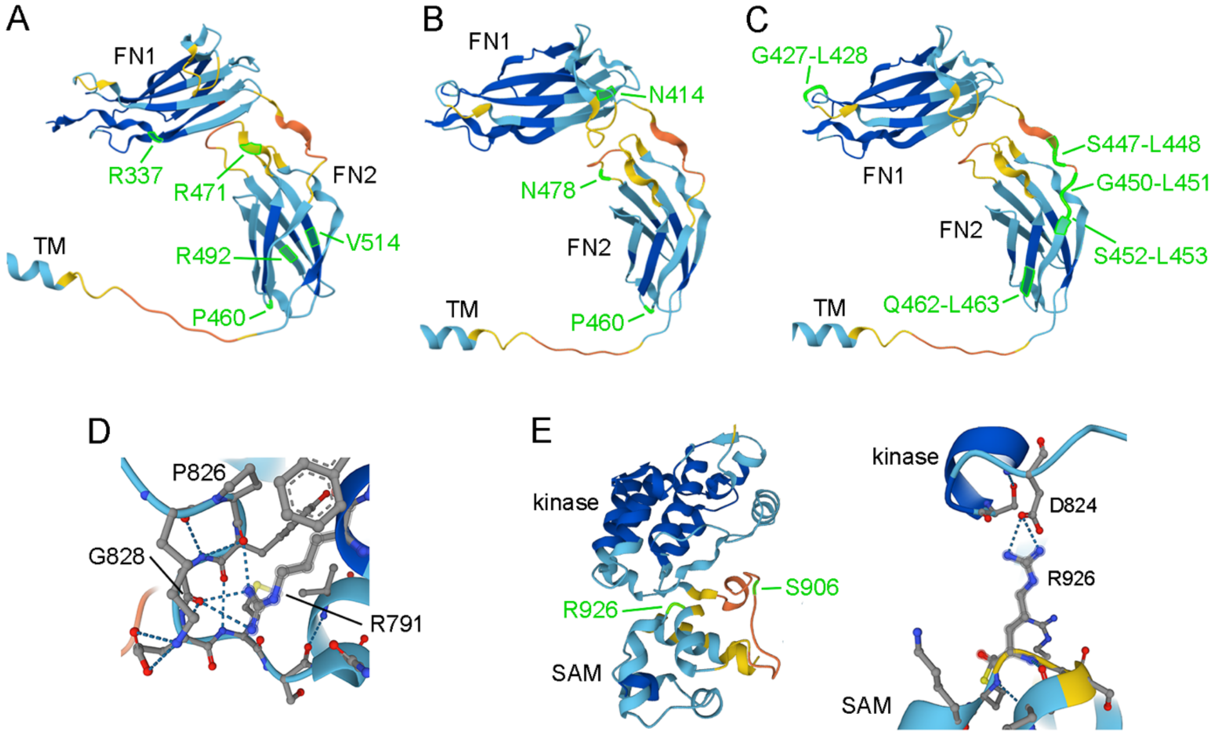

Figure S2. Location of EPHA1 residues of interest in a receptor structural model generated by AlphaFold. (**A**) EPHA1 residues in the fibronectin type III domains that have been identified as mutated in Alzheimer's patients. (**B**) Location of EPHA1 N-glycosylation sites relative to residue P460. (**C**) Location of predicted MMP cleavage sites in the fibronectin type III domains (see Fig. S4). (**D**) Close-up showing hydrogen bonds between the side chain of R791 and the backbone of P826 and G828. (**E**) Potential interaction between kinase and SAM domains through hydrogen bonds between the side chains of R926 in the SAM domain and D824 in the kinase domain. AlphaFold2 models are colored according to per-residue model confidence score (pLLDT) from high (> 90, dark blue) to very low (< 50, orange) pLDDT. Residues of interest are highlighted in green in the cartoon representations and indicated in green font. Key interacting residues are labeled in black. Hydrogen bonds are indicated as dotted lines. FN1, first fibronectin type III domain; FN2, second fibronectin type III domain; TM, transmembrane helix; kinase, kinase domain; SAM, SAM domain.

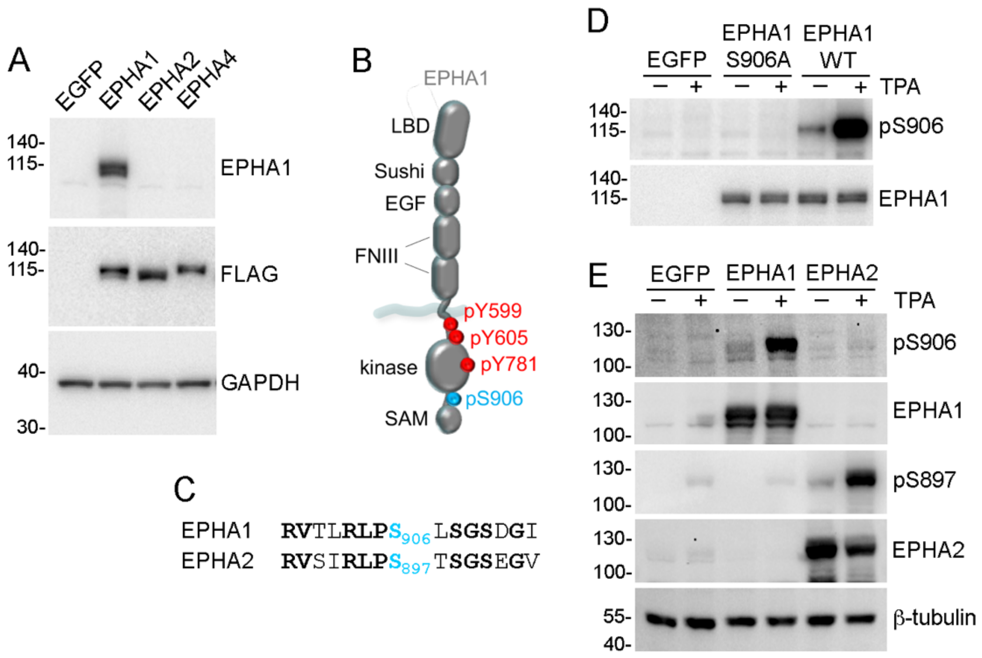

Figure S3. New antibodies specifically recognizing the EPHA1 SAM domain and the EPHA1 S906 phosphorylated motif. (**A**) A new rabbit polyclonal antibody (generated using the EPHA1 region including the kinase-SAM linker and the SAM domain as the antigen) specifically recognizes EPHA1, but not EPHA2 or EPHA4, in lysates of transiently transfected HEK293 cells. The Flag immunoblot demonstrates similar expression levels of the three Flag-tagged Eph receptors. (**B**) Schematic illustrating EPHA1 tyrosine phosphorylation and S906 phosphorylation sites. (**C**) Amino acid sequence surrounding EPHA1 S906 aligned with the sequence surrounding EPHA2 S897 shows the high similarity of the two phosphorylation motifs (identical amino acids are in bold). (**D**) The antibody recognizing the EPHA1 S906 phosphorylated motif does not label the EPHA1 S906A mutant (which lacks the S906 phosphorylation site) in transiently transfected HEK293 cells left untreated (–) or treated with 200 nM TPA for 30 min (+). (**E**) The antibody recognizing the phosphorylated S906 motif does not recognize the phosphorylated S897 motif of EPHA2 in transiently transfected HEK293 cells left untreated (–) or treated with 200 nM TPA for 30 min (+). Lysates in A, D, E were probed with the indicated antibodies; antibodies to GAPDH or β-tubulin were used to verify equal loading of the lanes.

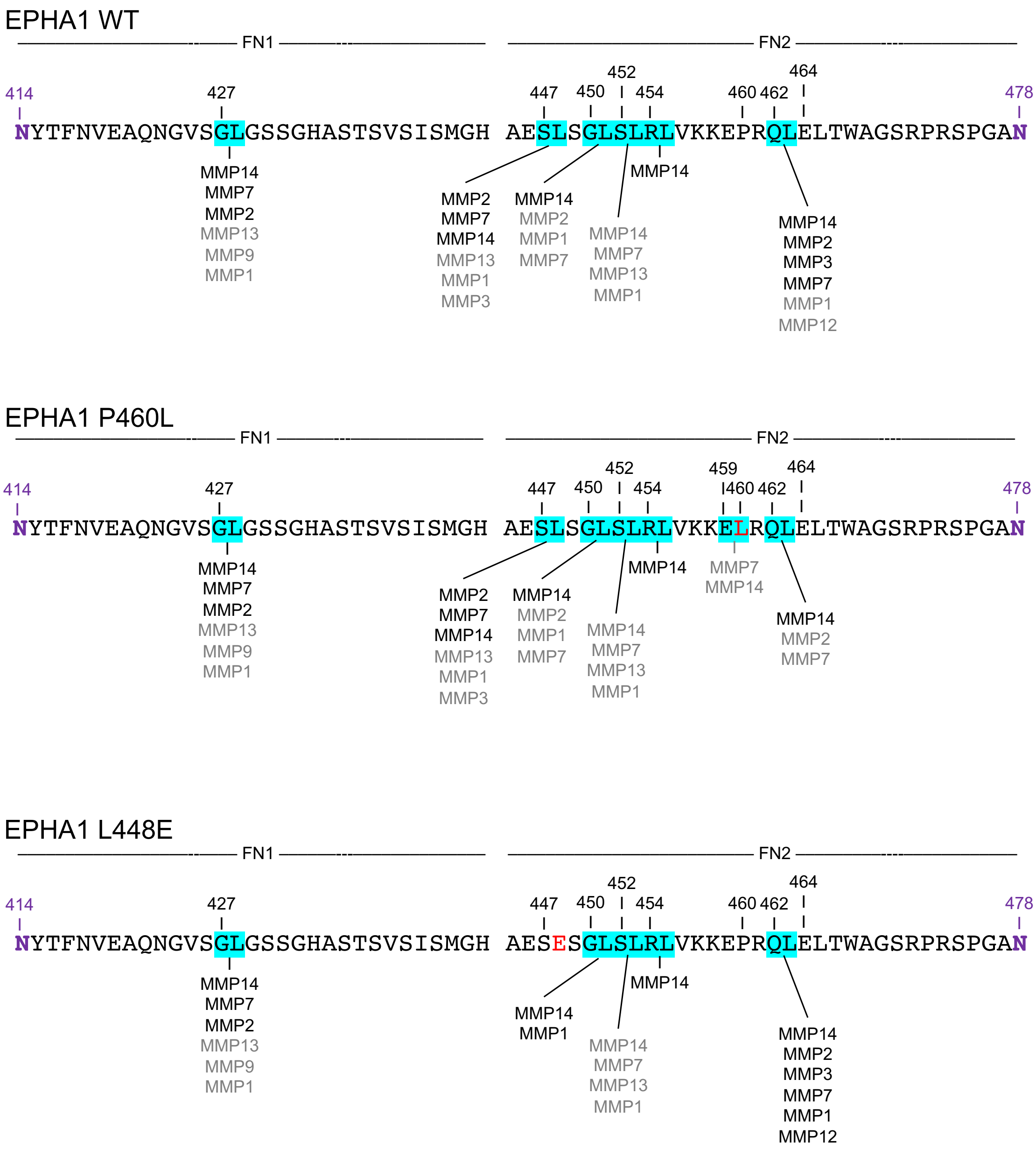

Figure S4. Predicted MMP cleavage sites in the EPHA1 extracellular region between the N414 and N478 glycosylation sites. Predicted MMP cleavage sites are highlighted in blue, with the residue preceding the cleavage labeled. L460 (in the P460L Alzheimer's mutant) and E448 (in the engineered L448E mutant) are shown in red font. The P460L mutation decreases the probability of cleavage between Q462 and L463 but creates a new potential cleavage site between E459 and L460. The L448E mutation disrupts the cleavage site after S447. Predictions of the most likely cleavage sites were made using the web resource ProsperousPlus (http://prosperousplus.unimelb-biotools.cloud.edu.au/). The MMPs with highest probability to cleave a particular site (score >0.9) are indicated in black font and the MMPs with still high but lower score (>0.8) are indicated in gray. Cleavage sites with at least 1 MMP scored >0.9 or at least 3 MMPs scored >0.8 were considered the most likely and are shown. The only exception is the E459-L460 site in the P460L mutant, which is predicted to be cleaved by 2 MMPs with score >0.8.
